# Clinical–Histopathological Concordance of Surgical Triage and the Unexpected Acute Phenotype in 773 Consecutive Laparoscopic Cholecystectomies

**DOI:** 10.3390/medicina62102003

**Published:** 2026-10-16

**Authors:** Bilal Aydın, Rifat Burak Ergül, Ahmet Barcın

**Affiliations:** 1 Department of General Surgery, Mus State Hospital, Mus 49200, Türkiye; drahmetbarcin@gmail.com; 2 Department of Urology, Mus State Hospital, Mus 49200, Türkiye; rifat-ergul@hotmail.com

**Keywords:** laparoscopic cholecystectomy, surgical triage, histopathology, acute cholecystitis, unexpected acute pathology, gallbladder wall thickening, C-reactive protein/albumin ratio

## Abstract

*Background and Objectives*: Surgical triage and final histopathological classification do not always fully overlap. This study evaluated their concordance, the frequency of unexpected acute pathology among electively scheduled patients, and associated preoperative characteristics. *Materials and Methods*: This single-center, single-surgeon retrospective cohort included 773 patients whose cholecystectomy was completed laparoscopically between 1 January 2022 and 30 June 2026. Surgical triage was classified as emergency or elective, and histopathology as acute or chronic inflammatory pathology. “Unexpected acute pathology” denoted acute histopathology in electively scheduled patients, irrespective of clinical suspicion. Multivariable logistic regression was performed in 627 elective patients with complete data. Model A included previous biliary attack and preoperative radiological gallbladder wall thickening; Model B additionally included the C-reactive protein/albumin ratio (CAR). *Results*: Of 773 patients, 138 underwent emergency and 635 elective surgery. Observed agreement was 92.4%, with Cohen’s kappa of 0.757 (95% bootstrap confidence interval: 0.697–0.814). Unexpected acute pathology occurred in 42 elective patients (6.6%). In Model B, previous biliary attack (odds ratio [OR] = 2.76), preoperative radiological gallbladder wall thickening (OR = 4.09), and CAR (OR = 1.52 per 5-unit increase) were associated with unexpected acute pathology. Adding CAR increased the area under the receiver operating characteristic curve from 0.697 to 0.756 and improved model fit (*p* = 0.00055). Unexpected acute pathology occurred in 3.8%, 11.9%, and 37.5% of patients with 0, 1, and 2 findings, respectively (n = 477, 126, and 24). Across these strata, documented omental adhesions increased from 23.5% to 62.5% and postoperative hospital stay >1 day from 9.2% to 41.7%. *Conclusions*: Surgical triage showed high overall concordance with histopathology. The two preoperative findings were associated with unexpected acute pathology and a more complex perioperative profile; CAR provided complementary information. Given the small two-finding subgroup and single-center design, external validation is required before these findings can guide operative planning.

## 1. Introduction

Laparoscopic cholecystectomy is one of the most commonly performed procedures in general surgery. The decision to perform surgery under emergency or elective conditions is based on the combined assessment of symptoms, physical examination findings, inflammatory markers, and imaging results. Early laparoscopic cholecystectomy has been shown in randomized trials and systematic reviews to be safe and effective in acute cholecystitis and to reduce overall treatment duration and hospital stay; accordingly, an early surgical approach has also been adopted in current international guidelines [1,2,3]. However, the extent to which real-world surgical triage corresponds to the final histopathological findings of the resected gallbladder has been less extensively investigated.

An important methodological issue arises when evaluating this relationship. The widely used Tokyo Guidelines for acute cholecystitis incorporate clinical findings, inflammatory markers such as leukocyte count and C-reactive protein, and imaging findings as part of the diagnostic framework [2]. Therefore, evaluating the performance of these same variables in predicting acute cholecystitis defined by criteria that already include them may introduce a degree of circularity. Histopathological examination, in contrast, allows direct postoperative assessment of inflammatory changes in the gallbladder independently of the preoperative clinical classification. Previous studies have demonstrated that preoperative clinical or radiological assessment does not fully correspond to final histopathological findings [4,5,6]. Accordingly, the relationship between surgical triage and histopathological classification should be approached as an assessment of concordance between two different levels of evaluation rather than as a conventional diagnostic accuracy problem.

A clinically important aspect of this discordance concerns patients who are scheduled electively but are found to have acute inflammatory changes on final histopathological examination. Although these patients enter the operating schedule as routine elective cases, their perioperative characteristics may differ from those of elective patients with chronic histopathology. A history of previous biliary attack, preoperative radiological gallbladder wall thickening, and inflammatory markers have previously been associated with more complex laparoscopic cholecystectomy, longer operative duration, or conversion to open surgery [7,8,9,10,11]. The C-reactive protein/albumin ratio (CAR) has also been evaluated as an inflammatory marker associated with operative course and conversion in acute cholecystitis [10,11]. However, the relationship of these readily available preoperative characteristics with unexpected acute pathology in electively scheduled patients, and the corresponding quantitative pathological morphology and perioperative profile of this phenotype, remain insufficiently defined.

The primary aim of this study was to evaluate concordance between surgical triage and final histopathological classification in a consecutive single-surgeon series of laparoscopic cholecystectomies and to determine the frequency of unexpected acute pathology among electively scheduled patients. The secondary aims were to investigate preoperative characteristics associated with unexpected acute pathology; to assess the additional information provided by adding CAR to a clinical model based on previous biliary attack and preoperative radiological gallbladder wall thickening; to compare concordant and discordant clinical–histopathological groups with respect to pathological gallbladder wall thickness and specimen morphology; and to examine the associations of the unexpected acute phenotype with operative duration, documented omental adhesions, postoperative hospital stay, and 30-day postoperative outcomes.

## 2. Materials and Methods

### 2.1. Study Design and Patient Selection

This single-center, single-surgeon retrospective observational cohort study included consecutive patients whose cholecystectomy was completed laparoscopically at Mus State Hospital between 1 January 2022 and 30 June 2026. Four procedures requiring conversion to open surgery were not part of the study dataset because the prespecified study population and ethics approval were limited to laparoscopic cholecystectomy. Two patients who underwent laparoscopic subtotal cholecystectomy were retained in the study cohort because the procedure remained laparoscopic and the cases fulfilled the study eligibility criteria. Demographic characteristics, comorbidities, American Society of Anesthesiologists (ASA) score, preoperative laboratory and radiological findings, operative records, histopathology reports, and 30-day postoperative follow-up data were obtained from the hospital information system and archived medical records.

Patients with adenocarcinoma or eosinophilic calculous cholecystitis on histopathological examination were excluded; all remaining cases were included in the analysis.

The study was approved by the Mus Alparslan University Scientific Research and Publication Ethics Committee (application date: 21 May 2026; meeting date: 16 June 2026; meeting no. 5; decision no. 72/Karar-65) and was conducted in accordance with the principles of the Declaration of Helsinki. Because of the retrospective design, the requirement for individual informed consent was waived by the ethics committee.

### 2.2. Surgical Triage and Histopathological Classification

Surgical triage was classified as emergency or elective according to the clinical setting in which surgery was performed. The emergency group consisted of patients who underwent surgery during the same hospital admission following presentation to the emergency department, whereas the elective group consisted of patients who were placed on a scheduled surgical list after outpatient evaluation and underwent surgery under elective conditions. This classification reflected routine clinical management decisions made by the surgeon independently of the aims of the present study.

Final histopathological classification was based on histopathology reports generated as part of routine clinical practice by the institutional pathology department. The acute inflammatory category included acute cholecystitis, acute-on-chronic cholecystitis, gangrenous cholecystitis, and emphysematous cholecystitis. The chronic inflammatory category included chronic cholecystitis and xanthogranulomatous cholecystitis. Xanthogranulomatous cholecystitis was classified within the chronic category because of its predominantly chronic inflammatory nature. The pathological wall-thickness threshold of ≥4 mm used in the morphological analyses was not used to define the acute or chronic histopathological categories.

Patients who were scheduled electively but demonstrated acute inflammatory pathology on final histopathological examination were defined throughout the study as having “unexpected acute pathology.” This definition does not imply that the elective surgical decision was incorrect or inappropriate; it describes only the discordance between preoperative surgical planning and final histopathological classification. In this context, “unexpected” refers to the postoperative histopathological finding relative to the preoperative surgical scheduling category and does not imply that acute inflammation was necessarily clinically unsuspected.

### 2.3. Preoperative Clinical, Radiological, and Laboratory Variables

Gallbladder wall thickness and gallbladder distension were evaluated based on findings reported in preoperative radiology reports. In elective patients, the ultrasonographic examination performed closest to the index operation was used, whereas imaging in emergency presentations was performed during the acute admission and consisted predominantly of computed tomography. Gallbladder wall status was coded as normal or thickened, and gallbladder size as normal or distended/hydropic, according to the interpretation documented in the routine radiology report. A single numerical wall-thickness cut-off was not imposed because quantitative wall measurements were not consistently reported across examinations. Because imaging modality was not coded as a separate and complete variable in the study dataset, no quantitative modality-specific comparison was performed.

A history of previous biliary attack was defined as a prior presentation to a healthcare facility or treatment for biliary colic, acute cholecystitis, biliary pancreatitis, or cholangitis. This information was retrospectively obtained from hospital records and patient charts; cases in which the history could not be reliably determined were treated as missing for this variable.

For laboratory analyses, blood samples obtained at the time of hospital admission following presentation to the emergency department were used for emergency patients, whereas samples obtained during the preoperative anesthesia assessment and premedication process were used for elective patients. In elective patients, blood sampling was typically performed 1–3 days before surgery, most often 1 day before the operation. Because sampling timing and clinical context differed between the two triage groups, the primary multivariable analysis involving the C-reactive protein/albumin ratio (CAR) was restricted to the elective cohort. CAR was calculated by dividing serum C-reactive protein expressed in mg/L by serum albumin expressed in g/dL. Detailed definitions and coding schemes for the study variables are provided in Supplementary Table S1.

### 2.4. Pathological Morphological Variables

The macroscopic dimensions and wall thickness of the gallbladder specimen were evaluated using measurements recorded in routine histopathology reports. Specimen size was recorded in two axes in millimeters, and the transverse diameter was used for morphological comparisons. Pathological wall thickness was analyzed directly in millimeters and was also dichotomized as <4 mm and ≥4 mm to assess quantitative morphological wall thickening. The institutional pathological wall classification comprised the categories ≤3 mm, 4–6 mm, 7–10 mm, and >10 mm, and the ≥4 mm definition was fully concordant with the numerical wall measurements.

Because gallbladder dimensions were not numerically reported in all preoperative ultrasonography reports, quantitative specimen measurements recorded in routine histopathology reports were used for between-group morphological comparisons. This approach was intended to provide a more standardized and comparable morphological assessment that was less affected by differences in imaging modality, operator, and reporting practices.

### 2.5. Operative and Postoperative Outcomes

Operative duration was assessed in minutes based on the times documented in operative records. Because operative times were routinely recorded in 5-min intervals in the hospital information system, durations in the dataset were expressed as multiples of 5 min.

Omental adhesions were considered present only when explicitly documented in the operative note and were recorded as “documented omental adhesions.” Cases without explicit documentation were not classified as having documented adhesions; therefore, this variable reflects retrospective documentation rather than a prospectively standardized assessment of adhesions.

Hospital stay was defined as the number of postoperative days from the index operation to discharge; preoperative hospitalization was not included in this calculation.

Postoperative complications were defined as events occurring within 30 days after surgery and were graded according to the Clavien–Dindo classification [12]. Thirty-day complications were identified retrospectively from postoperative follow-up information available in the hospital information system and archived medical records. Bile fistula, surgical site infection, postoperative pancreatitis, postoperative cholangitis/common bile duct stone, gallbladder-bed abscess or collection, bile duct injury, and postoperative hemorrhage were evaluated as separate complication categories.

### 2.6. Statistical Analysis

All analyses were performed using R version 4.6.1 (R Foundation for Statistical Computing, Vienna, Austria). Continuous variables were summarized as medians with 25th–75th percentiles. Comparisons between two independent groups were performed using the Mann–Whitney U test, and comparisons among three groups using the Kruskal–Wallis test. Categorical variables were presented as counts and percentages; Pearson’s chi-square test or Fisher’s exact test was used for group comparisons, as appropriate according to expected cell counts.

Concordance between surgical triage and final histopathological classification was assessed using the observed agreement rate and Cohen’s kappa coefficient. Cohen’s kappa was used to quantify agreement beyond chance between these two categorical classifications applied to the same patients. The 95% confidence interval for Cohen’s kappa was calculated using a percentile bootstrap method with 2000 resamples. Because surgical triage was not treated as a diagnostic screening test, measures of diagnostic accuracy such as sensitivity, specificity, positive predictive value, and negative predictive value were not included in the primary concordance analysis.

Preoperative factors associated with unexpected acute pathology in the elective cohort were examined using multivariable logistic regression. The models were constructed using a clinically justified parsimonious approach rather than data-driven univariable screening. Model A included a history of previous biliary attack and preoperative radiological gallbladder wall thickening. CAR was added in Model B to evaluate the incremental contribution of this biomarker to the baseline clinical model. No formal a priori sample-size or required event-count calculation was performed; study size was determined by the number of consecutive eligible patients during the defined study period. Given the 42 observed outcome events, model complexity was limited to two predictor parameters in Model A and three in Model B, excluding the intercept. The corresponding events-per-variable values of 21 and 14 were calculated from the observed data and were not used as an a priori sample-size criterion. Patients with missing information on previous biliary attack history were excluded from the relevant multivariable analyses using a complete-case approach.

Regression results were reported as odds ratios (ORs), 95% confidence intervals (CIs), and two-sided *p* values. To improve clinical interpretability, the effect of CAR was expressed per 5-unit increase; the corresponding OR was obtained by rescaling the fitted regression coefficient. Model discrimination was assessed using the area under the receiver operating characteristic curve (AUC). The conventional ROC framework was used for interpretation of AUC, and 95% CIs for AUC were estimated using a percentile bootstrap method with 2000 resamples, with the model refitted within each bootstrap sample [13,14]. Model performance was also assessed using the Brier score and Akaike information criterion (AIC) [15]. The nested models were compared using the likelihood-ratio test. To assess optimism within the development sample, bootstrap optimism correction of the AUC was performed using 500 resamples [16]. Model calibration was additionally assessed using the calibration intercept and calibration slope, with internal optimism correction based on 500 bootstrap resamples. Ideal calibration corresponds to an intercept of 0 and a slope of 1.

To evaluate the combined clinical information provided by previous biliary attack and preoperative radiological gallbladder wall thickening, patients were stratified according to the presence of 0, 1, or 2 of these findings. The graded associations between the number of findings and unexpected acute pathology, documented omental adhesions, and postoperative hospital stay >1 day were assessed using logistic regression; the association with operative duration was evaluated using Spearman rank correlation. The appropriateness of modeling the number of findings as a linear term was assessed by comparing categorical and linear specifications using likelihood-ratio tests.

The preoperative, pathological, operative, and postoperative characteristics of elective patients with unexpected acute pathology were compared with those of elective patients with chronic histopathology and emergency patients with acute histopathology. Associations of unexpected acute pathology with postoperative hospital stay >1 day and 30-day postoperative complications were additionally evaluated in logistic regression models adjusted for ASA score and age as sensitivity analyses.

Pathological gallbladder wall thickness and specimen transverse diameter were compared across clinical–histopathological subgroups. Pathological wall thickness was evaluated both as a continuous variable and dichotomized as <4 mm versus ≥4 mm. The association between preoperative radiological gallbladder wall thickening and pathological wall thickness ≥4 mm was assessed using Fisher’s exact test and univariable logistic regression.

Two additional sensitivity analyses were performed to assess the influence of acute-on-chronic cholecystitis on the main findings. In the first analysis, patients with acute-on-chronic cholecystitis were excluded from the analytical cohort; in the second, they were retained but reclassified into the chronic inflammatory pathology category.

The association between gangrenous cholecystitis and CAR was evaluated in an exploratory analysis. In this analysis, the 95% CI for AUC was estimated using 2000 bootstrap resamples, and the Youden index derived from the study dataset was reported as an exploratory cut-off. This value was not interpreted as a clinically validated decision threshold.

All tests were two-sided, and *p* < 0.05 was considered statistically significant. No adjustment was made for multiple comparisons. Accordingly, *p* values from secondary and exploratory analyses should be interpreted cautiously, and the corresponding findings should be regarded as hypothesis-generating rather than confirmatory. The models developed in this study were considered explanatory association models designed to evaluate factors associated with unexpected acute pathology and the incremental contribution of CAR to the baseline clinical model, rather than externally validated clinical prediction tools.

A language-model-based artificial intelligence tool was used during the development and review of the R analysis script; all code, analyses, and numerical outputs were independently checked and verified by the authors. The final R code enabling reproducibility of the analyses is provided as Supplementary Code.

## 3. Results

### 3.1. Patient Characteristics and Surgical Triage

During the study period, 780 consecutive patients underwent cholecystectomy completed laparoscopically. Four patients with adenocarcinoma and three with eosinophilic calculous cholecystitis were excluded, leaving 773 patients for the main analysis. The patient selection process and flow of the analysis cohorts are shown in Figure 1, while the detailed distribution of final histopathological diagnoses is presented in Supplementary Table S2. A history of previous biliary attack could not be determined in 11 patients (1.4%); no missing data were present for the other key preoperative and operative variables (Supplementary Table S3).

Of the 773 patients, 138 (17.9%) underwent emergency surgery and 635 (82.1%) underwent elective surgery. Patients in the emergency group were older than those in the elective group [51.0 (41.2–59.8) vs. 46.0 (36.0–59.0) years; *p* = 0.025], were more frequently male [58/138 (42.0%) vs. 153/635 (24.1%); *p* < 0.001], and more often had comorbidities [70/138 (50.7%) vs. 245/635 (38.6%); *p* = 0.009]. ASA class distribution also differed between the groups (*p* = 0.012). By contrast, previous biliary attack history was similar [24/135 (17.8%) vs. 118/627 (18.8%); *p* = 0.903]. Preoperative radiological gallbladder wall thickening was more frequent in the emergency group [127/138 (92.0%) vs. 56/635 (8.8%); *p* < 0.001]. CAR was also higher in the emergency group [13.1 (2.0–37.5) vs. 0.6 (0.2–1.3); *p* < 0.001]; however, because blood samples were obtained in different clinical contexts in the two groups, this comparison was interpreted descriptively (Table 1).

### 3.2. Concordance Between Surgical Triage and Histopathological Classification

Histopathological examination identified acute inflammatory pathology in 163 patients and chronic inflammatory pathology in 610 patients. Among the 138 patients who underwent emergency surgery, 121 (87.7%) had acute and 17 (12.3%) had chronic histopathology. Among the 635 patients who underwent elective surgery, 42 (6.6%) had acute and 593 (93.4%) had chronic histopathology.

All patients in the emergency-chronic group underwent surgery within the first 0–72 h after symptom onset. In the emergency-acute group, 90.9% underwent surgery within 0–72 h, 6.6% between 72 h and 1 week, and 2.5% between 7 and 10 days. The distribution of time from symptom onset to surgery did not differ between the two groups (*p* = 0.728).

CAR was lower in the emergency-chronic group than in the emergency-acute group [1.58 (1.11–3.19) vs. 16.3 (2.71–40.0); *p* < 0.001], and preoperative radiological gallbladder wall thickening was less frequent (76.5% vs. 94.2%; *p* = 0.031). Previous biliary attack history was similar between the groups (17.6% vs. 17.8%; *p* = 1.000).

Observed agreement between surgical triage and final histopathological classification was 92.4%. Cohen’s kappa was 0.757, with a 95% bootstrap confidence interval of 0.697–0.814. Unexpected acute pathology was identified in 42 elective patients (6.6%) (Table 2).

### 3.3. Factors Associated with Unexpected Acute Pathology in Elective Patients

Because information on previous biliary attack was missing for 8 patients in the elective cohort, the multivariable analysis was performed in 627 complete cases with 42 events. In Model A, a history of previous biliary attack (OR = 3.09; 95% CI: 1.57–6.10; *p* = 0.001) and preoperative radiological gallbladder wall thickening (OR = 4.88; 95% CI: 2.31–10.29; *p* < 0.001) were independently associated with unexpected acute pathology. In Model B, which additionally included CAR, previous biliary attack (OR = 2.76; 95% CI: 1.36–5.61; *p* = 0.005), preoperative radiological gallbladder wall thickening (OR = 4.09; 95% CI: 1.86–9.00; *p* < 0.001), and CAR (OR = 1.52 per 5-unit increase; 95% CI: 1.20–1.94; *p* < 0.001) were independently associated with unexpected acute pathology (Table 3).

The AUCs of Model A and Model B were 0.697 and 0.756, respectively, while the bootstrap optimism-corrected AUCs were 0.696 and 0.752. Model B had a lower AIC (272.57 vs. 282.50) and Brier score (0.0548 vs. 0.0576). The bootstrap optimism-corrected calibration intercepts were 0.012 and 0.009, and the corresponding calibration slopes were 1.013 and 0.942 for Models A and B, respectively. Addition of CAR significantly improved model fit (likelihood-ratio test, *p* = 0.00055). Detailed model comparison results are presented in Supplementary Table S4.

The contribution of CAR to individual predicted probabilities was more limited than that of the two basic clinical findings. Among patients with both a history of previous biliary attack and preoperative radiological gallbladder wall thickening, the predicted probability of unexpected acute pathology was 28.9% at the median CAR level and 32.6% at the 90th percentile (Supplementary Table S5).

### 3.4. Morphological and Perioperative Correlates of Unexpected Acute Pathology

Patients with unexpected acute pathology also differed from the elective-chronic group in quantitative pathological morphology. The transverse diameter of the gallbladder specimen was larger in the unexpected acute group than in the elective-chronic group [35 (26.2–40) vs. 30 (25–35) mm; *p* < 0.001], but smaller than in the emergency-acute group [35 (26.2–40) vs. 40 (36–45) mm; *p* < 0.001]. Pathological gallbladder wall thickness was greater in the unexpected acute group than in the elective-chronic group [5.5 (4–7.75) vs. 3 (2–3) mm; *p* < 0.001]. No statistically significant difference in pathological wall thickness was detected between the unexpected acute and emergency-acute groups [5.5 (4–7.75) vs. 6 (4–8) mm; *p* = 0.485].

Pathological wall thickness ≥4 mm was observed in 33/42 (78.6%) patients with unexpected acute pathology, 128/593 (21.6%) elective-chronic patients, and 101/121 (83.5%) emergency-acute patients. The unexpected acute group differed markedly from the elective-chronic group (*p* < 0.001), whereas no significant difference was observed compared with the emergency-acute group (*p* = 0.488) (Table 4A).

A significant association was also observed between preoperative radiological gallbladder wall thickening and pathological wall thickness. In the elective cohort, pathological wall thickness ≥4 mm was present in 23.0% of patients without radiological wall thickening and in 50.0% of those with radiological wall thickening. Preoperative radiological gallbladder wall thickening was associated with higher odds of pathological wall thickness ≥4 mm (OR = 3.35; 95% CI: 1.91–5.88; *p* < 0.001).

Among elective patients, operative duration was longer in those with unexpected acute pathology than in those with chronic histopathology [40 (30–40) vs. 25 (25–30) min; *p* < 0.001]. Documented omental adhesions were more frequent [27/42 (64.3%) vs. 143/593 (24.1%); *p* < 0.001], and postoperative hospital stay >1 day was also more common [16/42 (38.1%) vs. 63/593 (10.6%); *p* < 0.001].

Operative duration was similar between the unexpected acute and emergency-acute groups [40 (30–40) vs. 40 (30–40) min; *p* = 0.663]. Documented omental adhesions occurred in 27/42 (64.3%) and 58/121 (47.9%) patients, respectively (*p* = 0.075). Postoperative hospital stay >1 day occurred in 16/42 (38.1%) patients in the unexpected acute group and 83/121 (68.6%) in the emergency-acute group (*p* < 0.001).

The 30-day complication rate was 4/42 (9.5%) in the unexpected acute group and 21/593 (3.5%) in the elective-chronic group (*p* = 0.076). Clavien–Dindo grade ≥III complications occurred in 2/42 (4.8%) and 8/593 (1.3%), respectively (*p* = 0.137) (Table 4B).

After adjustment for age and ASA score, the association between unexpected acute pathology and 30-day postoperative complications did not reach statistical significance (OR = 2.77; 95% CI: 0.83–9.26; *p* = 0.097). In contrast, the association with postoperative hospital stay >1 day remained significant after adjustment (OR = 6.63; 95% CI: 3.04–14.45; *p* < 0.001) (Supplementary Table S6).

Overall, 47 patients (6.1%) developed a postoperative complication within 30 days. The detailed distribution of complication types and Clavien–Dindo grades is presented in Supplementary Table S7. Three patients (0.4%) sustained bile duct injuries requiring intervention; two occurred in the emergency-acute group and one in the elective-chronic group, whereas no bile duct injury occurred in the unexpected acute group (Supplementary Table S8).

### 3.5. Clinical Stratification Using Two Simple Preoperative Findings

When previous biliary attack and preoperative radiological gallbladder wall thickening were considered together, 477 of 627 elective patients had neither finding, 126 had one finding, and 24 had both findings. The frequency of unexpected acute pathology was 3.8%, 11.9%, and 37.5%, respectively. With each additional finding, the odds of unexpected acute pathology increased 3.80-fold (95% CI: 2.42–5.97; *p* < 0.001).

Documented omental adhesions occurred in 23.5%, 33.3%, and 62.5% of patients across the 0-, 1-, and 2-finding groups, respectively, while postoperative hospital stay >1 day occurred in 9.2%, 19.8%, and 41.7%. For each additional finding, the OR was 1.95 for documented omental adhesions (95% CI: 1.43–2.67; *p* < 0.001) and 2.57 for postoperative hospital stay >1 day (95% CI: 1.77–3.72; *p* < 0.001). Operative duration also increased with the number of findings [25 (25–30), 30 (25–30), and 40 (30–40) min; Spearman ρ = 0.268; *p* < 0.001] (Table 5).

When the analysis was restricted to 585 electively scheduled patients with chronic final histopathology and complete data for both preoperative findings, a similar graded association persisted. Operative duration increased with the number of findings (Spearman ρ = 0.202; *p* < 0.001). For each additional finding, the OR was 1.49 for documented omental adhesions (95% CI: 1.03–2.14; *p* = 0.033) and 2.27 for postoperative hospital stay >1 day (95% CI: 1.46–3.53; *p* < 0.001) (Table 6).

### 3.6. Sensitivity Analyses

The main associations persisted after patients with acute-on-chronic cholecystitis were excluded from the analysis. In Model B, previous biliary attack (OR = 3.06; 95% CI: 1.35–6.93; *p* = 0.007), preoperative radiological gallbladder wall thickening (OR = 4.29; 95% CI: 1.75–10.57; *p* = 0.002), and CAR (OR = 1.61 per 5-unit increase; 95% CI: 1.26–2.06; *p* < 0.001) remained significantly associated with unexpected acute pathology. The AUCs of Model A and Model B were 0.719 and 0.798, respectively; addition of CAR significantly improved model fit (likelihood-ratio test, *p* = 0.00017) (Supplementary Table S9).

In the second sensitivity analysis, in which the same cases were reclassified into the chronic inflammatory pathology category, addition of CAR again significantly improved model fit (AIC: 225.96 vs. 213.89; likelihood-ratio test, *p* = 0.00018) (Supplementary Table S10).

### 3.7. Exploratory Analysis of Gangrenous Cholecystitis

Among the 163 patients with acute inflammatory pathology, 26 (16.0%) had gangrenous cholecystitis. CAR was higher in patients with gangrenous cholecystitis than in those with other acute inflammatory pathologies [21.2 (9.7–52.2) vs. 5.1 (1.4–32.9); *p* = 0.003]. The AUC of CAR for discriminating gangrenous cholecystitis was 0.684 (95% CI: 0.588–0.777). The data-derived Youden cut-off was 5.03, with a sensitivity of 88.5% and specificity of 49.6%. This analysis was exploratory, and the identified value was not interpreted as a clinical decision threshold (Supplementary Table S11).

## 4. Discussion

The principal finding of this study is that real-world surgical triage showed high overall concordance with final histopathological classification, while discordant subgroups were identified in both the emergency and elective cohorts. Most patients undergoing emergency surgery had acute inflammatory pathology, whereas most electively scheduled patients had chronic inflammatory pathology; overall observed agreement was 92.4%, with a Cohen’s kappa of 0.757. Nevertheless, unexpected acute pathology was present in 6.6% of elective patients. Compared with elective-chronic patients, this subgroup had longer operative duration, more frequent documented omental adhesions and postoperative hospital stay >1 day, and different pathological morphological characteristics.

Incomplete correspondence between clinical assessment and final histopathological diagnosis has been reported previously [4,5,6]. The diagnosis of acute cholecystitis integrates symptoms, systemic inflammatory findings, and imaging, as reflected in the Tokyo Guidelines [2]. Histopathology should therefore not be regarded as a “gold standard” for determining whether a clinical management decision was correct or incorrect. In this study, histopathology served as a postoperative comparator for assessing correspondence between surgical scheduling and inflammatory classification, rather than the appropriateness of treatment. The term “unexpected acute pathology” was used operationally to describe acute histopathology in electively scheduled patients and does not imply that inflammation was clinically unsuspected.

The emergency-chronic subgroup illustrates this distinction. All such patients underwent surgery within the first 0–72 h after symptom onset, and the vast majority of patients in the emergency-acute group were operated on within the same interval; no statistically significant difference in surgical timing was detected between the groups. The recorded timing therefore did not support delayed surgery as the sole explanation for chronic histopathology. CAR levels were markedly lower and preoperative radiological gallbladder wall thickening was less frequent in the emergency-chronic group, whereas previous biliary attack history was similar. These findings suggest that an acute symptomatic presentation does not necessarily correspond to acute histopathological inflammation. They should not be interpreted as evidence that emergency surgery was unnecessary in patients with chronic histopathology.

Within the elective cohort, previous biliary attack and preoperative radiological gallbladder wall thickening were associated with unexpected acute pathology in multivariable analysis. When these findings were considered together, the frequency of unexpected acute pathology increased from 3.8% in patients with neither finding to 11.9% in those with one finding and 37.5% in those with both findings. Previous biliary events and gallbladder wall thickening have previously been associated with a more complex operative course [7,8,9]. Our findings suggest that these features are also associated with histopathologically active inflammation in electively scheduled patients.

Pathological morphology provided a quantitative correlate of the unexpected acute subgroup. Specimen transverse diameter was greater than in elective-chronic patients but smaller than in emergency-acute patients. Pathological wall thickness was greater than in elective-chronic patients, whereas no statistically significant difference was detected compared with emergency-acute patients. Wall thickness ≥4 mm was present in 78.6% of unexpected acute patients, compared with 21.6% of elective-chronic and 83.5% of emergency-acute patients. In the elective cohort, pathological wall thickness ≥4 mm was also more frequent in patients with radiological wall thickening than in those without it (50.0% vs. 23.0%; OR = 3.35), providing a link between preoperative radiological findings and specimen morphology. These postoperative measurements provide structural context for the histopathological classification but should not be regarded as preoperative decision variables.

The associations with these two preoperative findings extended beyond histopathological classification. As the number of findings increased, operative duration, documented omental adhesions, and the rate of postoperative hospital stay >1 day increased in a graded manner. In the subgroup with both findings, documented omental adhesions occurred in 62.5%, postoperative hospital stay >1 day occurred in 41.7%, and the median operative duration was 40 min. However, this two-feature stratification should be regarded as exploratory and hypothesis-generating rather than as a validated clinical risk-stratification tool. In particular, estimates for the subgroup with both findings (n = 24) should be interpreted cautiously as descriptive findings. Importantly, similar directional associations persisted when the analysis was restricted to elective patients with chronic final histopathology. This pattern suggests that the two findings may reflect the persistent operative effects of prior inflammation, adhesions, or fibrotic change, rather than acute histopathology alone.

These findings suggest that routinely available preoperative information may help characterize differences in the perioperative profiles of elective patients. However, the usefulness of this stratification for guiding operative planning requires prospective assessment and external validation. Documented omental adhesions should not be equated with surgical difficulty because they were identified retrospectively from operative notes rather than through standardized prospective grading.

The addition of CAR provided statistically significant incremental information to the model. The AUC increased from 0.697 in Model A to 0.756 in Model B after CAR was added; AIC and Brier scores also improved, and the likelihood-ratio test supported this incremental contribution [13,14,15,16]. The bootstrap optimism-corrected calibration slope of 0.942 for Model B was close to 1, although calibration in independent populations remains to be established. CAR has previously been associated with a more complex operative course and conversion to open surgery in acute cholecystitis [10,11]. The present study extends this association to unexpected acute histopathology within the elective cohort. The contribution of CAR should nevertheless be interpreted in the context of the range examined. When previous biliary attack and preoperative radiological gallbladder wall thickening were held constant, increasing CAR from the median to the 90th percentile produced only a limited absolute change in predicted probability. Within this cohort, these results support considering CAR as a complementary inflammatory biomarker rather than a substitute for clinical and radiological information.

The association between unexpected acute pathology and postoperative hospital stay >1 day persisted after adjustment for age and ASA score. Although the 30-day complication rate was numerically higher than in elective-chronic patients, the difference was not statistically significant; no significant difference was observed in Clavien–Dindo grade ≥III morbidity either [12]. These findings support an association with longer postoperative hospitalization, but conclusions regarding postoperative morbidity remain limited by the small number of events.

The influence of acute-on-chronic classification was examined by excluding these cases from one sensitivity analysis and reclassifying them as chronic in another. Adding CAR improved model fit in both analyses, suggesting that its incremental contribution was not solely explained by including acute-on-chronic cholecystitis in the acute inflammatory category.

In the exploratory analysis, CAR levels were markedly higher in patients with gangrenous cholecystitis than in those with other acute inflammatory pathologies. The AUC of CAR for discriminating gangrenous cholecystitis was 0.684, and the data-derived cut-off of 5.03 showed high sensitivity but limited specificity. These results should therefore be regarded as exploratory rather than as evidence supporting a clinical decision threshold. The three patients with emphysematous cholecystitis were included among the non-gangrenous acute inflammatory pathologies; no separate analysis was performed because of the small number of cases.

The strengths of this study include the use of a consecutive patient series, performance of all operations by a single surgeon, surgical triage determined under real-world clinical conditions independently of the study aims, and histopathological classification based on routine final pathology reports. The single-surgeon setting reduced inter-operator variability, while quantitative specimen measurements allowed the clinical–histopathological groups to be compared beyond their diagnostic labels. Clinically motivated parsimonious models, together with assessment of discrimination, calibration, overall performance, and bootstrap optimism correction, provided a structured internal evaluation of the models [13,14,15,16].

This study has several limitations. Its retrospective, single-center, single-surgeon design limits external generalizability. Surgical triage was based on routine clinical decisions rather than a prospectively standardized algorithm; therefore, the observed concordance and associations may reflect local patient characteristics, imaging practices, and the surgeon’s thresholds for emergency versus elective surgery. The contribution of individual components of the triage decision could not be quantified. The exclusion of the four patients who required conversion to open surgery may have resulted in underrepresentation of the most technically difficult cases. Consequently, the findings may not be generalizable to the full spectrum of patients undergoing cholecystectomy, particularly those requiring conversion.

Laboratory samples were obtained at different clinical time points in elective and emergency patients, limiting direct CAR comparisons between the two groups; accordingly, the primary multivariable CAR models were restricted to the elective cohort. Because imaging modality was not completely coded as a separate variable, the individual performance of ultrasonography and computed tomography could not be evaluated. Histopathological morphological measurements were derived from routine reports rather than prospectively standardized study-specific measurements. Omental adhesions and 30-day postoperative complications were identified retrospectively from available clinical records. The absence of explicit documentation of adhesions does not establish their absence, and under-documentation may have led to underestimation of their frequency. Incomplete ascertainment of postoperative complications also cannot be excluded.

Previous biliary attack history was missing in 11 patients, including eight in the elective cohort. Although the proportion of missing data was small, the complete-case approach may have introduced selection bias if patients with unavailable histories differed systematically from those retained in the analyses. The primary multivariable models were deliberately parsimonious because of the limited number of outcome events; however, residual confounding by demographic, comorbidity-related, or other unmeasured clinical factors cannot be excluded. Given the number of secondary analyses and the absence of adjustment for multiple comparisons, secondary *p* values should be interpreted cautiously. In view of these limitations and the absence of external validation, the models should be interpreted as explanatory association models describing factors related to the unexpected acute phenotype rather than as independent clinical decision tools.

## 5. Conclusions

In conclusion, surgical triage and final histopathological classification showed high overall concordance but did not fully overlap. In elective patients, a history of previous biliary attack and preoperative radiological gallbladder wall thickening were associated with unexpected acute pathology, while CAR provided complementary information to the clinical model. Pathological wall thickness in the unexpected acute subgroup was greater than in elective-chronic patients and numerically close to that observed in emergency-acute patients, providing a quantitative morphological correlate of this subgroup. The same two simple preoperative findings were also associated with longer operative duration, a higher frequency of documented omental adhesions, and postoperative hospital stay >1 day. Given the retrospective, single-center, single-surgeon design and the small subgroup with both findings, these associations require external validation before they can be used to guide operative planning. These findings do not constitute a validated clinical decision tool or imply that the original surgical triage decisions were inappropriate.

## Figures and Tables

**Figure 1:**
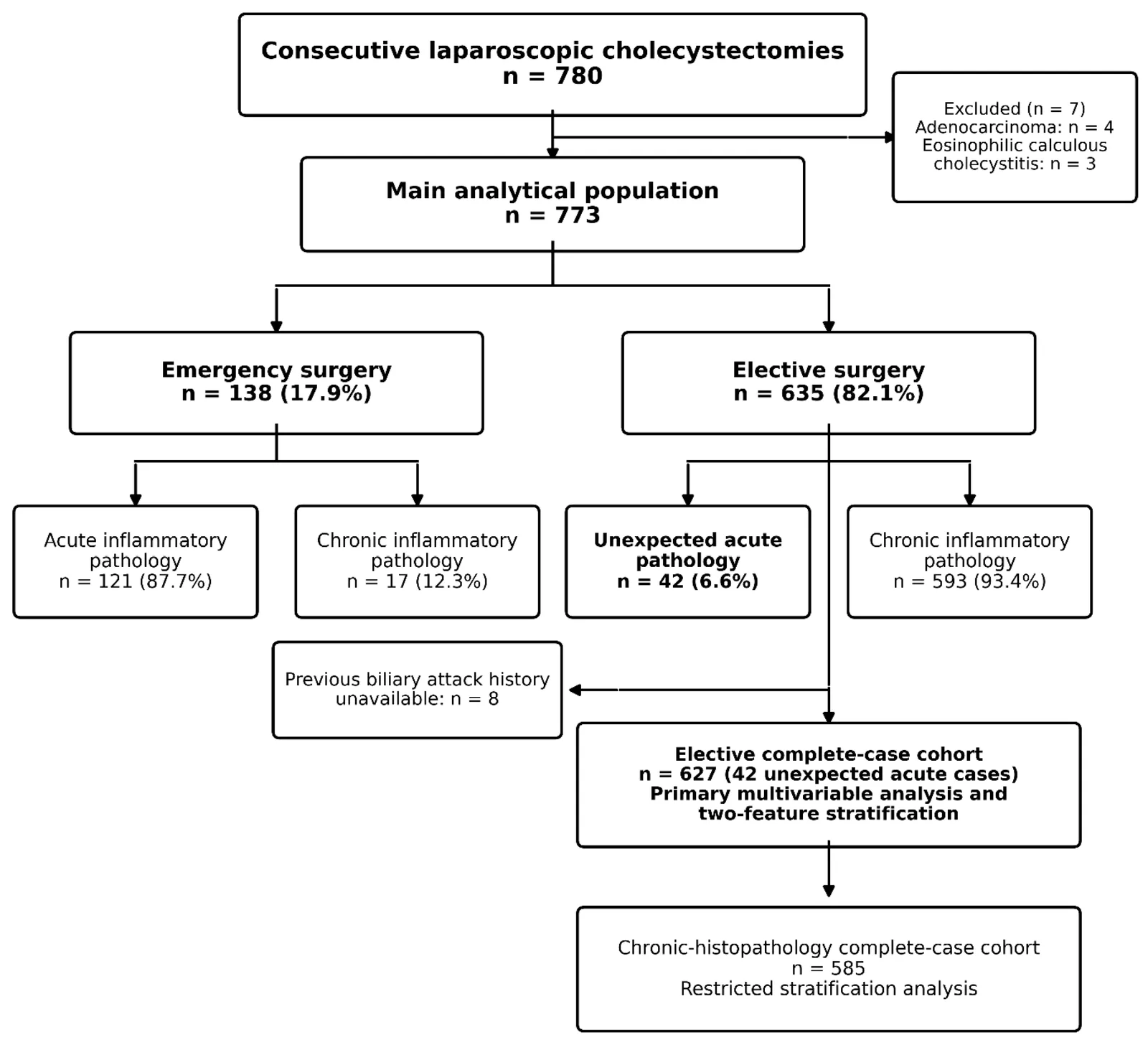
Flow diagram of patient selection and analysis cohorts. Among 780 consecutive patients who underwent laparoscopic cholecystectomy during the study period, 4 patients with adenocarcinoma and 3 patients with eosinophilic calculous cholecystitis were excluded, leaving 773 patients for the main analysis. Of these, 138 underwent emergency surgery and 635 underwent elective surgery. In the elective cohort, previous biliary attack history was unavailable in 8 patients; therefore, the primary multivariable analysis and two-feature stratification were performed in 627 complete cases, including 42 patients with unexpected acute pathology. The restricted stratification analysis among elective patients with chronic histopathology included 585 complete cases.

**Table 1:** Baseline demographic and clinical characteristics according to surgical triage.

Variable	Emergency (n = 138)	Elective (n = 635)	*p* Value
Age, years	51.0 (41.2–59.8)	46.0 (36.0–59.0)	0.025
Male sex	58/138 (42.0%)	153/635 (24.1%)	<0.001
ASA score	2.0 (1.0–2.0)	1.0 (1.0–2.0)	0.022
ASA class, n (%)			0.012
ASA I	68 (49.3%)	389 (61.3%)	
ASA II	64 (46.4%)	210 (33.1%)	
ASA III	6 (4.3%)	36 (5.7%)	
Presence of comorbidity	70/138 (50.7%)	245/635 (38.6%)	0.009
Number of comorbidities	1.0 (0.0–1.0)	0.0 (0.0–1.0)	0.018
History of previous biliary attack	24/135 (17.8%)	118/627 (18.8%)	0.903
Preoperative radiological gallbladder wall thickening	127/138 (92.0%)	56/635 (8.8%)	<0.001
C-reactive protein/albumin ratio	13.1 (2.0–37.5)	0.6 (0.2–1.3)	<0.001

Data are presented as median (25th–75th percentile) or n/N (%), as appropriate. Continuous variables were compared using the Mann–Whitney U test, and categorical variables using Pearson’s chi-square test or Fisher’s exact test, as appropriate. The *p* value for ASA class represents the overall comparison of the ASA I–III distribution. Blood samples were obtained in different clinical contexts in emergency and elective patients; therefore, the between-group comparison of the C-reactive protein/albumin ratio should be interpreted descriptively.

**Table 2:** Concordance between surgical triage and final histopathological classification. (a) Distribution of histopathological categories according to surgical triage; (b) Concordance measures and discordant subgroup frequencies.

(a)			
**Surgical triage**	**Acute inflammatory pathology, n (%)**	**Chronic inflammatory pathology, n (%)**	**Total**
Emergency	121 (87.7%)	17 (12.3%)	138
Elective	42 (6.6%)	593 (93.4%)	635
Total	163 (21.1%)	610 (78.9%)	773
(b)			
**Concordance measure**			**Value**
Observed agreement			92.4%
Cohen’s kappa			0.757
95% bootstrap CI			0.697–0.814
Unexpected acute pathology among elective patients			42/635 (6.6%)
Chronic histopathology among emergency patients			17/138 (12.3%)

The acute inflammatory category included acute cholecystitis, acute-on-chronic cholecystitis, gangrenous cholecystitis, and emphysematous cholecystitis. The chronic inflammatory category included chronic cholecystitis and xanthogranulomatous cholecystitis. Cohen’s kappa was calculated for agreement between surgical triage and histopathological classification. Because surgical triage represents a clinical management decision rather than a diagnostic screening test, diagnostic accuracy measures such as sensitivity, specificity, positive predictive value, and negative predictive value were not reported.

**Table 3:** Factors associated with unexpected acute pathology in the elective cohort. (A) Multivariable associations; (B) Model performance.

(A)				
**Variable**	**Model A OR (95% CI)**	***p*** **value**	**Model B OR (95% CI)**	***p** * **value**
Previous biliary attack	3.09 (1.57–6.10)	0.001	2.76 (1.36–5.61)	0.005
Preoperative radiological gallbladder wall thickening	4.88 (2.31–10.29)	<0.001	4.09 (1.86–9.00)	<0.001
C-reactive protein/albumin ratio, per 5-unit increase	—	—	1.52 (1.20–1.94)	<0.001
(B)				
**Measure**			**Model A**	**Model B**
Patients/events			627/42	627/42
Events per variable			21.0	14.0
AUC			0.697	0.756
Bootstrap 95% CI for AUC			0.607–0.780	0.673–0.838
Optimism-corrected AUC			0.696	0.752
AIC			282.50	272.57
Brier score			0.0576	0.0548

OR, odds ratio; CI, confidence interval; CAR, C-reactive protein/albumin ratio; AUC, area under the receiver operating characteristic curve; AIC, Akaike information criterion. Model A included previous biliary attack and preoperative radiological gallbladder wall thickening. Model B additionally included CAR. Odds ratios, 95% CIs, and *p* values in Panel A were estimated using multivariable logistic regression. The effect of CAR is presented per 5-unit increase by rescaling the fitted regression coefficient to improve clinical interpretability. Addition of CAR significantly improved model fit compared with Model A (likelihood-ratio test, *p* = 0.00055). AUC confidence intervals were estimated using 2000 bootstrap resamples, with the model refitted within each bootstrap sample. Internal validation was performed using bootstrap optimism correction with 500 resamples. The models are presented as association models rather than externally validated clinical prediction tools.

**Table 4:** Characteristics and outcomes of the unexpected acute phenotype. (A) Preoperative and pathological characteristics; (B) Operative and postoperative outcomes.

(A)					
**Variable**	**Unexpected acute (n = 42)**	**Elective-chronic (n = 593)**	***p** * value *	**Emergency-acute (n = 121)**	***p** * **value †**
Gallbladder wall thickening	14/42 (33.3%)	42/593 (7.1%)	<0.001	114/121 (94.2%)	<0.001
Distended/hydropic gallbladder	4/42 (9.5%)	13/593 (2.2%)	0.021	109/121 (90.1%)	<0.001
Previous biliary attack	19/42 (45.2%)	99/585 (16.9%)	<0.001	21/118 (17.8%)	<0.001
C-reactive protein/albumin ratio	1.3 (0.5–4.6)	0.6 (0.2–1.2)	<0.001	16.3 (2.7–40.0)	<0.001
Gallbladder specimen transverse diameter, mm	35.0 (26.2–40.0)	30.0 (25.0–35.0)	<0.001	40.0 (36.0–45.0)	<0.001
Pathological gallbladder wall thickness, mm	5.5 (4–7.75)	3 (2–3)	<0.001	6 (4–8)	0.485
Pathological gallbladder wall thickness ≥4 mm	33/42 (78.6%)	128/593 (21.6%)	<0.001	101/121 (83.5%)	0.488
Time on elective waiting list, months	2.0 (0.2–8.8)	2.0 (1.0–19.0)	0.591	—	—
(B)					
**Variable**	**Unexpected acute (n = 42)**	**Elective-chronic (n = 593)**	***p** * value *	**Emergency-acute (n = 121)**	***p** * **value †**
Operative time, min	40 (30–40)	25 (25–30)	<0.001	40 (30–40)	0.663
Documented omental adhesions	27/42 (64.3%)	143/593 (24.1%)	<0.001	58/121 (47.9%)	0.075
Postoperative hospital stay >1 day	16/42 (38.1%)	63/593 (10.6%)	<0.001	83/121 (68.6%)	<0.001
30-day complication	4/42 (9.5%)	21/593 (3.5%)	0.076	21/121 (17.4%)	0.321
Clavien–Dindo grade ≥ III	2/42 (4.8%)	8/593 (1.3%)	0.137	5/121 (4.1%)	1.000

Data are presented as median (25th–75th percentile) or n/N (%), as appropriate. * Comparison between unexpected acute and elective-chronic patients. † Comparison between unexpected acute and emergency-acute patients. Continuous variables were compared using the Mann–Whitney U test and categorical variables using Fisher’s exact test. Comparisons of the C-reactive protein/albumin ratio between elective and emergency groups should be interpreted descriptively because blood samples were obtained in different clinical contexts. Gallbladder wall thickening and gallbladder distension/hydrops refer to preoperative radiological findings.

**Table 5:** Stratification by two simple preoperative findings in the elective cohort. (a) Distribution of clinical and perioperative outcomes according to the number of findings; (b) Associations per additional preoperative finding.

(a)					
**Number of findings**	**n**	**Unexpected acute pathology, n/N (%)**	**Operative time, min**	**Documented omental adhesions, n/N (%)**	**Postoperative hospital stay >1 day, n/N (%)**
0	477	18/477 (3.8%)	25 (25–30)	112/477 (23.5%)	44/477 (9.2%)
1	126	15/126 (11.9%)	30 (25–30)	42/126 (33.3%)	25/126 (19.8%)
2	24	9/24 (37.5%)	40 (30–40)	15/24 (62.5%)	10/24 (41.7%)
(b)					
**Outcome**		**OR per additional finding (95% CI)**			***p** * **value**
Unexpected acute pathology		3.80 (2.42–5.97)			<0.001
Documented omental adhesions		1.95 (1.43–2.67)			<0.001
Postoperative hospital stay >1 day		2.57 (1.77–3.72)			<0.001

The two preoperative findings were a history of previous biliary attack and preoperative radiological gallbladder wall thickening. The number of findings was treated as an ordinal variable because categorical specification did not provide superior model fit for unexpected acute pathology (*p* = 0.729), documented omental adhesions (*p* = 0.210), or postoperative hospital stay >1 day (*p* = 0.788). Operative time increased with the number of findings (Spearman ρ = 0.268, *p* < 0.001). This two-feature stratification is exploratory and hypothesis-generating, not a validated clinical risk-stratification tool. Estimates for the subgroup with both findings (n = 24) should be interpreted cautiously.

**Table 6:** Stratification analysis restricted to elective patients with chronic histopathology. (a) Operative and postoperative characteristics according to the number of findings; (b) Associations per additional preoperative finding.

(a)				
**Number of findings**	**n**	**Operative time, min**	**Documented omental adhesions, n/N (%)**	**Postoperative hospital stay >1 day, n/N (%)**
0	459	25 (25–30)	103/459 (22.4%)	40/459 (8.7%)
1	111	30 (25–30)	33/111 (29.7%)	17/111 (15.3%)
2	15	30 (30–40)	6/15 (40.0%)	6/15 (40.0%)
(b)				
**Outcome**		**OR per additional finding (95% CI)**		***p** * **value**
Documented omental adhesions		1.49 (1.03–2.14)		0.033
Postoperative hospital stay >1 day		2.27 (1.46–3.53)		<0.001

This analysis was restricted to electively scheduled patients whose final histopathology was classified as chronic inflammatory pathology. Operative time remained positively associated with the number of findings (Spearman ρ = 0.202, *p* < 0.001). The overall Fisher’s exact test for documented omental adhesions across the three strata did not reach statistical significance (*p* = 0.092), despite the significant ordinal association.

## Data Availability

Anonymized study data are available from the corresponding author upon reasonable and justified request, subject to ethical and institutional restrictions. The final R code enabling reproducibility of the analyses is provided as Supplementary Code.

## Supplementary Materials

The following supporting information can be downloaded at https://www.mdpi.com/article/10.3390/medicina62102003/s1, Supplementary Table S1. Variable coding dictionary. Supplementary Table S2. Study population and histopathological diagnosis distribution. Supplementary Table S3. Missing-data summary. Supplementary Table S4. Detailed comparison of Model A and Model B in the primary elective cohort. Supplementary Table S5. Predicted probability of unexpected acute pathology according to CAR level and two binary clinical findings. Supplementary Table S6. Sensitivity analyses of postoperative outcomes adjusted for ASA score and age. Supplementary Table S7. Detailed distribution of postoperative complications. Supplementary Table S8. Major bile duct injuries requiring intervention. Supplementary Table S9. Sensitivity analysis excluding acute-on-chronic cholecystitis. Supplementary Table S10. Sensitivity analysis reclassifying acute-on-chronic cholecystitis into the chronic inflammatory pathology category. Supplementary Table S11. Exploratory performance of CAR for identifying gangrenous cholecystitis. Supplementary Code. Final R code enabling reproducibility of the statistical analyses.
