# Supplementary material for: Clinical–Histopathological Concordance of Surgical Triage and the Unexpected Acute Phenotype in 773 Consecutive Laparoscopic Cholecystectomies
Source: Medicina (Kaunas). 2026 Oct 16;62(10):2003. doi: 10.3390/medicina62102003 (PMC13707155; doi:10.3390/medicina62102003)
Supplement: Supplementary file 1 [file medicina-62-02003-s001.zip › Supplementary Code.pdf]

Supplementary Code

```
# ============================================================
# Supplementary Code
# Clinical–Histopathological Concordance of Surgical Triage
# and the Unexpected Acute Phenotype in 773 Consecutive
# Laparoscopic Cholecystectomies
#
# R version used for the manuscript analyses: 4.6.1
# ============================================================

# ------------------------------------------------------------
# 1. Packages
# ------------------------------------------------------------
library(readxl)
library(dplyr)
library(boot)
library(pROC)

# ------------------------------------------------------------
# 2. Import de-identified source data
# ------------------------------------------------------------
# Select the cleaned, de-identified Excel workbook when prompted.
data_file <- file.choose()
dat_raw <- read_excel(data_file)

# Initial dimensions and source-variable checks
dim(dat_raw)
names(dat_raw)

table(dat_raw$`ANA HİSTOPATOLOJİK TANI`, useNA = "ifany")
table(dat_raw$`ACİL/ELEKTİF O: ACİL/1 ELEKTİF`, useNA = "ifany")
table(dat_raw$`OPERASYON ÖNCESİ ATAK`, useNA = "ifany")
table(dat_raw$`radyoloji duvar`, useNA = "ifany")

# ------------------------------------------------------------
# 3. Construct analysis dataset
# ------------------------------------------------------------
dat <- dat_raw %>%
  transmute(
    id = `SIRA NO`,
    sex = `CİNSİYET 0: KADIN 1: ERKEK`,
    age = `YAŞ(yıl)`,

    # The source workbook contained three entries coded as "ht";
    # these patients had HT=1 and were therefore coded as having
    # at least one comorbidity.
    comorbidity = case_when(
      trimws(tolower(as.character(`EK HASTALIK 0:YOK 1: VAR`))) == "0" ~ 0,
      trimws(tolower(as.character(`EK HASTALIK 0:YOK 1: VAR`))) == "1" ~ 1,
      trimws(tolower(as.character(`EK HASTALIK 0:YOK 1: VAR`))) == "ht" ~ 1,
      TRUE ~ NA_real_
    ),

    asa = `ASA SKORU`,
    triage = `ACİL/ELEKTİF O: ACİL/1 ELEKTİF`,
    radiol_wall = `radyoloji duvar`,
    radiol_size = `radyoloji KESE BOYUTU`,
    path_wall_mm = `patoloji DUVAR(mm)`,
    path_gb_size = as.character(`patoloji KESE BOYUTU(mm)`),
    histology_code = `ANA HİSTOPATOLOJİK TANI`,
    car = `CRP/ALBUMİN (CAR)`,
    complication = `KOMPLİKASYON VAR/YOK`,
    complication_type = `KOMPLİKASYON TÜRÜ`,
    complication_treatment = `KOMPLİKASYON TEDAVİSİ`,
    clavien = `CLAVIEN-DINDO`,
    operative_time = `AMELİYAT SÜRESİ (dk)`,
    omental_adhesion = `OMENTAL YAPIŞIKLIK`,
    previous_attack_raw = `OPERASYON ÖNCESİ ATAK`,
    elective_wait_months = `ELEKTİF SÜRE (ay)`,
    acute_interval = `AKUT SÜRE (saat)`,
    hospital_stay = `YATIŞ SÜRESİ (gün)`
  ) %>%
```

```
  mutate(
    previous_attack = case_when(
      previous_attack_raw == 0 ~ 0,
      previous_attack_raw == 1 ~ 1,
      previous_attack_raw == 9 ~ NA_real_,
      TRUE ~ NA_real_
    ),

    wall_thickening = case_when(
      radiol_wall == 1 ~ 0,
      radiol_wall == 2 ~ 1,
      TRUE ~ NA_real_
    ),

    gb_distended = case_when(
      radiol_size == 1 ~ 0,
      radiol_size == 2 ~ 1,
      TRUE ~ NA_real_
    ),

    acute_pathology = case_when(
      histology_code %in% c(1, 3, 4, 5) ~ 1,
      histology_code %in% c(2, 7) ~ 0,
      TRUE ~ NA_real_
    ),

    chronic_pathology = case_when(
      histology_code %in% c(2, 7) ~ 1,
      histology_code %in% c(1, 3, 4, 5) ~ 0,
      TRUE ~ NA_real_
    ),

    excluded = histology_code %in% c(6, 8),

    unexpected_acute = case_when(
      triage == 1 & acute_pathology == 1 ~ 1,
      triage == 1 & acute_pathology == 0 ~ 0,
      triage == 0 ~ 0,
      TRUE ~ NA_real_
    ),

    path_wall_ge4 = ifelse(path_wall_mm >= 4, 1, 0),
    postop_stay_gt1 = ifelse(hospital_stay > 1, 1, 0),

    elective_wait_months_num = suppressWarnings(
      as.numeric(
        gsub(
          ",",
          ".",
          trimws(as.character(elective_wait_months))
        )
      )
    ),

    clavien_ge3 = ifelse(clavien %in% c(3, 5), 1, 0)
  )

# Primary analytical cohort:
# histology code 6 = adenocarcinoma
# histology code 8 = eosinophilic calculous cholecystitis
analysis <- dat %>%
  filter(!excluded)

# ------------------------------------------------------------
# 4. Primary cohort checks
# ------------------------------------------------------------
cat("Raw patients:", nrow(dat_raw), "\n")
cat("Analytical patients:", nrow(analysis), "\n\n")

cat("Histopathology codes:\n")
print(table(analysis$histology_code, useNA = "ifany"))

cat("\nAcute/chronic classification:\n")
```

```
print(table(analysis$acute_pathology, useNA = "ifany"))

cat("\nEmergency/elective:\n")
print(table(analysis$triage, useNA = "ifany"))

cat("\nUnexpected acute:\n")
print(table(analysis$unexpected_acute, useNA = "ifany"))

cat("\nPrevious biliary attack:\n")
print(table(analysis$previous_attack, useNA = "ifany"))

# -------------------------------------------------------------
# 5. Baseline comparisons (Table 1)
# -------------------------------------------------------------
# Continuous variables
wilcox.test(age ~ triage, data = analysis, exact = FALSE)
wilcox.test(asa ~ triage, data = analysis, exact = FALSE)
wilcox.test(car ~ triage, data = analysis, exact = FALSE)

# Categorical variables
chisq.test(table(analysis$triage, analysis$sex), correct = FALSE)
chisq.test(table(analysis$triage, analysis$comorbidity), correct = FALSE)
chisq.test(table(analysis$triage, analysis$asa))
fisher.test(table(analysis$triage, analysis$previous_attack))
fisher.test(table(analysis$triage, analysis$wall_thickening))

# -------------------------------------------------------------
# 6. Concordance between surgical triage and histopathology
# -------------------------------------------------------------
# Emergency is aligned with the acute side; elective with the chronic side.
triage_class <- ifelse(analysis$triage == 0, 1, 0)
path_class <- analysis$acute_pathology

observed_agreement <- mean(triage_class == path_class)

p1_triage <- mean(triage_class == 1)
p0_triage <- mean(triage_class == 0)
p1_path <- mean(path_class == 1)
p0_path <- mean(path_class == 0)

expected_agreement <-
  (p1_triage * p1_path) + (p0_triage * p0_path)

kappa_value <-
  (observed_agreement - expected_agreement) /
  (1 - expected_agreement)

c(
  observed_agreement = observed_agreement,
  cohens_kappa = kappa_value
)

table(
  Surgical_Triage = analysis$triage,
  Acute_Pathology = analysis$acute_pathology
)

# Percentile bootstrap 95% CI for Cohen's kappa
# Final reproducible manuscript value: 0.697-0.814
kappa_fun <- function(data, indices) {
  d <- data[indices, ]

  triage_class_b <- ifelse(d$triage == 0, 1, 0)
  path_class_b <- d$acute_pathology

  po <- mean(triage_class_b == path_class_b)

  p1_t <- mean(triage_class_b == 1)
  p0_t <- mean(triage_class_b == 0)
  p1_p <- mean(path_class_b == 1)
  p0_p <- mean(path_class_b == 0)

  pe <- (p1_t * p1_p) + (p0_t * p0_p)
```

```
  (po - pe) / (1 - pe)
}

set.seed(2026)

boot_kappa <- boot::boot(
  data = analysis,
  statistic = kappa_fun,
  R = 2000
)

kappa_ci <- quantile(
  boot_kappa$t,
  probs = c(0.025, 0.975),
  na.rm = TRUE
)

c(
  observed_agreement = observed_agreement,
  cohens_kappa = kappa_value,
  lower_95_CI = kappa_ci[1],
  upper_95_CI = kappa_ci[2]
)

# ------------------------------------------------------------
# 7. Emergency-chronic versus emergency-acute
# ------------------------------------------------------------
emergency_sub <- analysis %>%
  filter(triage == 0, acute_pathology %in% c(0, 1))

table(
  Histopathology = emergency_sub$acute_pathology,
  Symptom_to_surgery = emergency_sub$acute_interval
)

fisher.test(
  table(
    emergency_sub$acute_pathology,
    emergency_sub$acute_interval
  )
)

wilcox.test(
  car ~ acute_pathology,
  data = emergency_sub,
  exact = FALSE
)

fisher.test(
  table(
    emergency_sub$acute_pathology,
    emergency_sub$wall_thickening
  )
)

fisher.test(
  table(
    emergency_sub$acute_pathology,
    emergency_sub$previous_attack
  )
)

# ------------------------------------------------------------
# 8. Primary elective multivariable models
# ------------------------------------------------------------
elective_cc <- analysis %>%
  filter(
    triage == 1,
    !is.na(previous_attack),
    !is.na(wall_thickening),
    !is.na(car),
    !is.na(unexpected_acute)
```

```
  )

c(
  patients = nrow(elective_cc),
  events = sum(elective_cc$unexpected_acute)
)

fit_A <- glm(
  unexpected_acute ~ previous_attack + wall_thickening,
  data = elective_cc,
  family = binomial
)

fit_B <- glm(
  unexpected_acute ~ previous_attack + wall_thickening + car,
  data = elective_cc,
  family = binomial
)

summary(fit_A)
summary(fit_B)

# Wald ORs and 95% CIs
OR_A <- exp(cbind(
  OR = coef(fit_A),
  lower = coef(fit_A) - 1.96 * sqrt(diag(vcov(fit_A))),
  upper = coef(fit_A) + 1.96 * sqrt(diag(vcov(fit_A)))
))

OR_B <- exp(cbind(
  OR = coef(fit_B),
  lower = coef(fit_B) - 1.96 * sqrt(diag(vcov(fit_B))),
  upper = coef(fit_B) + 1.96 * sqrt(diag(vcov(fit_B)))
))

OR_A
OR_B

# CAR effect per 5-unit increase
beta_car <- coef(fit_B)["car"]
se_car <- sqrt(vcov(fit_B)["car", "car"])

CAR_5unit <- c(
  OR = exp(5 * beta_car),
  lower_95_CI = exp(5 * (beta_car - 1.96 * se_car)),
  upper_95_CI = exp(5 * (beta_car + 1.96 * se_car)),
  p_value = summary(fit_B)$coefficients["car", "Pr(>|z|)"]
)

CAR_5unit

# ------------------------------------------------------------
# 9. Model performance
# ------------------------------------------------------------
pred_A <- predict(fit_A, type = "response")
pred_B <- predict(fit_B, type = "response")

roc_A <- pROC::roc(
  response = elective_cc$unexpected_acute,
  predictor = pred_A,
  quiet = TRUE
)

roc_B <- pROC::roc(
  response = elective_cc$unexpected_acute,
  predictor = pred_B,
  quiet = TRUE
)

auc_A <- as.numeric(pROC::auc(roc_A))
auc_B <- as.numeric(pROC::auc(roc_B))

c(
```

```
  AUC_Model_A = auc_A,
  AUC_Model_B = auc_B,
  AIC_Model_A = AIC(fit_A),
  AIC_Model_B = AIC(fit_B)
)

# Likelihood-ratio test
anova(fit_A, fit_B, test = "LRT")

# Brier scores
brier_A <- mean(
  (elective_cc$unexpected_acute - pred_A)^2
)

brier_B <- mean(
  (elective_cc$unexpected_acute - pred_B)^2
)

c(
  Brier_Model_A = brier_A,
  Brier_Model_B = brier_B
)

# -------------------------------------------------------------
# 10. Percentile bootstrap 95% CIs for AUC
# Final reproducible values: Model A 0.607-0.780; Model B 0.673-0.838
#     The model is refitted within each bootstrap resample.
# -------------------------------------------------------------
auc_boot_fun <- function(data, indices, model_formula) {
  d <- data[indices, ]

  # Extremely rare one-class resamples are not informative for ROC AUC.
  if (length(unique(d$unexpected_acute)) < 2) {
    return(NA_real_)
  }

  fit <- glm(
    model_formula,
    data = d,
    family = binomial
  )

  pred <- predict(
    fit,
    newdata = d,
    type = "response"
  )

  roc_obj <- pROC::roc(
    response = d$unexpected_acute,
    predictor = pred,
    quiet = TRUE
  )

  as.numeric(pROC::auc(roc_obj))
}

set.seed(2026)

boot_auc_A <- boot::boot(
  data = elective_cc,
  statistic = function(data, indices) {
    auc_boot_fun(
      data,
      indices,
      unexpected_acute ~ previous_attack + wall_thickening
    )
  },
  R = 2000
)

boot_auc_B <- boot::boot(
  data = elective_cc,
```

```
  statistic = function(data, indices) {
    auc_boot_fun(
      data,
      indices,
      unexpected_acute ~ previous_attack + wall_thickening + car
    )
  },
  R = 2000
)

CI_A <- quantile(
  boot_auc_A$t,
  c(0.025, 0.975),
  na.rm = TRUE
)

CI_B <- quantile(
  boot_auc_B$t,
  c(0.025, 0.975),
  na.rm = TRUE
)

CI_A
CI_B

# ------------------------------------------------------------
# 11. Bootstrap optimism correction for AUC
# Final reproducible values: optimism 0.001/0.004; corrected AUC 0.696/0.752
# ------------------------------------------------------------
optimism_auc <- function(data, formula, B = 500) {

  fit_original <- glm(
    formula,
    data = data,
    family = binomial
  )

  pred_original <- predict(
    fit_original,
    newdata = data,
    type = "response"
  )

  auc_original <- as.numeric(
    pROC::auc(
      pROC::roc(
        data$unexpected_acute,
        pred_original,
        quiet = TRUE
      )
    )
  )

  optimism <- numeric(B)

  for (b in seq_len(B)) {

    idx <- sample(
      seq_len(nrow(data)),
      replace = TRUE
    )

    boot_data <- data[idx, ]

    if (length(unique(boot_data$unexpected_acute)) < 2) {
      optimism[b] <- NA_real_
      next
    }

    fit_boot <- glm(
      formula,
      data = boot_data,
      family = binomial
```

```
    )

    pred_boot <- predict(
      fit_boot,
      newdata = boot_data,
      type = "response"
    )

    auc_boot <- as.numeric(
      pROC::auc(
        pROC::roc(
          boot_data$unexpected_acute,
          pred_boot,
          quiet = TRUE
        )
      )
    )

    pred_test <- predict(
      fit_boot,
      newdata = data,
      type = "response"
    )

    auc_test <- as.numeric(
      pROC::auc(
        pROC::roc(
          data$unexpected_acute,
          pred_test,
          quiet = TRUE
        )
      )
    )

    optimism[b] <- auc_boot - auc_test
  }

  mean_optimism <- mean(
    optimism,
    na.rm = TRUE
  )

  c(
    apparent_auc = auc_original,
    optimism = mean_optimism,
    optimism_corrected_auc =
      auc_original - mean_optimism
  )
}

set.seed(2026)

opt_A <- optimism_auc(
  elective_cc,
  unexpected_acute ~ previous_attack + wall_thickening,
  B = 500
)

opt_B <- optimism_auc(
  elective_cc,
  unexpected_acute ~ previous_attack + wall_thickening + car,
  B = 500
)

opt_A
opt_B

# ------------------------------------------------------------
# 11A. Bootstrap optimism correction for calibration
#      (Supplementary Table S4)
# ------------------------------------------------------------
# Requires elective_cc, fit_A, and fit_B from the existing script.
# This section does not import the workbook again.
```

```
required_objects <- c("elective_cc", "fit_A", "fit_B")
missing_objects <- required_objects[
  !vapply(required_objects, exists, logical(1), inherits = TRUE)
]
if (length(missing_objects) > 0L) {
  stop("Run the preceding script sections first. Missing objects: ",
       paste(missing_objects, collapse = ", "))
}

calibration_variables <- c(
  "unexpected_acute", "previous_attack", "wall_thickening", "car"
)
if (!all(calibration_variables %in% names(elective_cc))) {
  stop("Required calibration variables are missing from elective_cc.")
}
if (anyNA(elective_cc[, calibration_variables, drop = FALSE])) {
  stop("The calibration cohort must have complete model data.")
}
if (nrow(elective_cc) != 627L ||
    sum(elective_cc$unexpected_acute) != 42L) {
  stop("Cohort differs from the manuscript: expected 627 patients and 42 events.")
}

formula_A <- formula(fit_A)
formula_B <- formula(fit_B)

calibration_stats <- function(y, linear_predictor) {
  # Calibration-in-the-large: slope fixed at 1; ideal intercept = 0.
  fit_intercept <- glm(
    y ~ 1,
    offset = linear_predictor,
    family = binomial()
  )

  # Calibration slope: intercept and slope jointly estimated.
  # Ideal slope = 1.
  fit_slope <- glm(
    y ~ linear_predictor,
    family = binomial()
  )

  if (!isTRUE(fit_intercept$converged) ||
      !isTRUE(fit_slope$converged)) {
    stop("A calibration regression did not converge.")
  }

  c(
    intercept = unname(coef(fit_intercept)[1]),
    slope = unname(coef(fit_slope)["linear_predictor"])
  )
}

bootstrap_calibration <- function(
    data, model_formula, R = 500L, seed = 2026L
) {
  # Preserve the random-number state used elsewhere in the script.
  old_seed <- get0(".Random.seed", envir = .GlobalEnv, inherits = FALSE)
  on.exit({
    if (is.null(old_seed)) {
      if (exists(".Random.seed", envir = .GlobalEnv, inherits = FALSE)) {
        rm(list = ".Random.seed", envir = .GlobalEnv)
      }
    } else {
      assign(".Random.seed", old_seed, envir = .GlobalEnv)
    }
  }, add = TRUE)
  set.seed(seed)

  n <- nrow(data)
  fit_original <- glm(model_formula, data = data, family = binomial())
  if (!isTRUE(fit_original$converged) ||
      any(!is.finite(coef(fit_original)))) {
```

```
  stop("The original model is not a finite, converged fit.")
}
lp_original <- predict(fit_original, newdata = data, type = "link")
original_cal <- calibration_stats(data$unexpected_acute, lp_original)

results <- matrix(
  NA_real_, nrow = R, ncol = 4L,
  dimnames = list(NULL, c(
    "intercept_apparent_boot", "intercept_test_original",
    "slope_apparent_boot", "slope_test_original"
  ))
)

for (b in seq_len(R)) {
  idx <- sample.int(n, size = n, replace = TRUE)
  boot_data <- data[idx, , drop = FALSE]
  if (length(unique(boot_data$unexpected_acute)) < 2L) next

  results[b, ] <- tryCatch({
    fit_boot <- glm(model_formula, data = boot_data, family = binomial())
    if (!isTRUE(fit_boot$converged) ||
        any(!is.finite(coef(fit_boot)))) {
      stop("Nonconverged or nonfinite bootstrap fit.")
    }

    lp_boot_apparent <- predict(
      fit_boot, newdata = boot_data, type = "link"
    )
    lp_boot_test <- predict(fit_boot, newdata = data, type = "link")

    cal_boot_apparent <- calibration_stats(
      boot_data$unexpected_acute, lp_boot_apparent
    )
    cal_boot_test <- calibration_stats(
      data$unexpected_acute, lp_boot_test
    )

    c(
      cal_boot_apparent["intercept"], cal_boot_test["intercept"],
      cal_boot_apparent["slope"], cal_boot_test["slope"]
    )
  }, error = function(e) rep(NA_real_, 4L))
}

valid <- apply(results, 1L, function(x) all(is.finite(x)))
if (!any(valid)) stop("No valid bootstrap calibration results.")
if (sum(valid) < R) {
  warning(sum(!valid), " bootstrap replicates were invalid; report the valid count.")
}
res_valid <- results[valid, , drop = FALSE]

optimism_intercept <- mean(
  res_valid[, "intercept_apparent_boot"] -
    res_valid[, "intercept_test_original"]
)
optimism_slope <- mean(
  res_valid[, "slope_apparent_boot"] -
    res_valid[, "slope_test_original"]
)

list(
  n_boot_requested = R,
  n_boot_valid = sum(valid),
  seed = seed,
  apparent_intercept = unname(original_cal["intercept"]),
  apparent_slope = unname(original_cal["slope"]),
  optimism_intercept = optimism_intercept,
  optimism_slope = optimism_slope,
  optimism_corrected_intercept =
    unname(original_cal["intercept"] - optimism_intercept),
  optimism_corrected_slope =
    unname(original_cal["slope"] - optimism_slope),
  bootstrap_details = results
```

```
  )
}

# Reset to the same seed for each model, as in the calibration analysis.
cal_A <- bootstrap_calibration(elective_cc, formula_A, R = 500L, seed = 2026L)
cal_B <- bootstrap_calibration(elective_cc, formula_B, R = 500L, seed = 2026L)

calibration_table <- data.frame(
  Measure = c(
    "Apparent calibration intercept",
    "Apparent calibration slope",
    "Bootstrap optimism: calibration intercept",
    "Bootstrap optimism: calibration slope",
    "Optimism-corrected calibration intercept",
    "Optimism-corrected calibration slope"
  ),
  Model_A = c(
    cal_A$apparent_intercept, cal_A$apparent_slope,
    cal_A$optimism_intercept, cal_A$optimism_slope,
    cal_A$optimism_corrected_intercept, cal_A$optimism_corrected_slope
  ),
  Model_B = c(
    cal_B$apparent_intercept, cal_B$apparent_slope,
    cal_B$optimism_intercept, cal_B$optimism_slope,
    cal_B$optimism_corrected_intercept, cal_B$optimism_corrected_slope
  )
)

cat("\nCALIBRATION COHORT AND VALID REPLICATES\n")
cat("Patients:", nrow(elective_cc),
    "| Events:", sum(elective_cc$unexpected_acute), "\n")
cat("Model A:", cal_A$n_boot_valid, "/", cal_A$n_boot_requested, "valid\n")
cat("Model B:", cal_B$n_boot_valid, "/", cal_B$n_boot_requested, "valid\n")

cat("\nCALIBRATION TABLE\n")
print(calibration_table, digits = 6, row.names = FALSE)

calibration_table_rounded <- calibration_table
calibration_table_rounded$Model_A <- round(calibration_table$Model_A, 3)
calibration_table_rounded$Model_B <- round(calibration_table$Model_B, 3)
cat("\nCALIBRATION TABLE (ROUNDED)\n")
print(calibration_table_rounded, row.names = FALSE)

# -------------------------------------------------------------
# 12. Predicted probabilities (Supplementary Table S5)
# -------------------------------------------------------------
car_quantiles <- quantile(
  elective_cc$car,
  probs = c(0.50, 0.75, 0.90),
  na.rm = TRUE
)

car_quantiles

pred_grid <- expand.grid(
  previous_attack = c(0, 1),
  wall_thickening = c(0, 1),
  car = as.numeric(car_quantiles)
)

pred_grid$predicted_probability <- predict(
  fit_B,
  newdata = pred_grid,
  type = "response"
)

pred_grid$CAR_level <- rep(
  c(
    "Median",
    "75th percentile",
    "90th percentile"
  ),
  each = 4
```

```
)

pred_grid$Previous_attack <- ifelse(
  pred_grid$previous_attack == 1,
  "Yes",
  "No"
)

pred_grid$Wall_thickening <- ifelse(
  pred_grid$wall_thickening == 1,
  "Yes",
  "No"
)

pred_grid$Predicted_probability_percent <-
  100 * pred_grid$predicted_probability

pred_grid[
  ,
  c(
    "Previous_attack",
    "Wall_thickening",
    "CAR_level",
    "car",
    "Predicted_probability_percent"
  )
]

# -------------------------------------------------------------
# 13. Clinical-histopathological subgroups
# -------------------------------------------------------------
analysis <- analysis %>%
  mutate(
    group3 = case_when(
      triage == 1 & acute_pathology == 1 ~ "Unexpected acute",
      triage == 1 & acute_pathology == 0 ~ "Elective-chronic",
      triage == 0 & acute_pathology == 1 ~ "Emergency-acute",
      TRUE ~ "Emergency-chronic"
    )
  )

table(analysis$group3)

UA <- analysis %>%
  filter(group3 == "Unexpected acute")

EC <- analysis %>%
  filter(group3 == "Elective-chronic")

EA <- analysis %>%
  filter(group3 == "Emergency-acute")

# -------------------------------------------------------------
# 14. Parse pathological gallbladder specimen dimensions
# -------------------------------------------------------------
# Source format: longitudinal x transverse dimension in millimeters,
# e.g. "88x20". The transverse (second) measurement is used.
analysis <- analysis %>%
  mutate(
    path_gb_size_clean = trimws(path_gb_size),

    path_gb_longitudinal = ifelse(
      grepl(
        "^[0-9]+[xX][0-9]+$",
        path_gb_size_clean
      ),
      as.numeric(
        sub(
          "[xX].*$",
          "",
          path_gb_size_clean
        )
      ),
```

```
      NA_real_
    ),

    path_gb_transverse = ifelse(
      grepl(
        "^[0-9]+[xX][0-9]+$",
        path_gb_size_clean
      ),
      as.numeric(
        sub(
          "^.*[xX]",
          "",
          path_gb_size_clean
        )
      ),
      NA_real_
    )
  )

# Verify that all analytical values have valid dimension format.
malformed_path_size <- analysis %>%
  filter(
    !is.na(path_gb_size_clean),
    path_gb_size_clean != "",
    is.na(path_gb_transverse)
  ) %>%
  select(
    id,
    path_gb_size
  )

print(malformed_path_size)

if (nrow(malformed_path_size) > 0) {
  stop(
    "Malformed pathological gallbladder size values remain. ",
    "Please verify the source workbook before analysis."
  )
}

# Recreate subgroup objects after adding parsed morphology
UA <- analysis %>%
  filter(group3 == "Unexpected acute")

EC <- analysis %>%
  filter(group3 == "Elective-chronic")

EA <- analysis %>%
  filter(group3 == "Emergency-acute")

# -----------------------------------------------------------
# 15. Morphological summaries and comparisons
# -----------------------------------------------------------
morph_summary <- analysis %>%
  filter(
    group3 %in% c(
      "Unexpected acute",
      "Elective-chronic",
      "Emergency-acute"
    )
  ) %>%
  group_by(group3) %>%
  summarise(
    n = n(),

    transverse_n =
      sum(!is.na(path_gb_transverse)),

    transverse_median =
      median(path_gb_transverse, na.rm = TRUE),

    transverse_q1 =
      quantile(path_gb_transverse, 0.25, na.rm = TRUE),
```

```
    transverse_q3 =
      quantile(path_gb_transverse, 0.75, na.rm = TRUE),

    wall_n =
      sum(!is.na(path_wall_mm)),

    wall_median =
      median(path_wall_mm, na.rm = TRUE),

    wall_q1 =
      quantile(path_wall_mm, 0.25, na.rm = TRUE),

    wall_q3 =
      quantile(path_wall_mm, 0.75, na.rm = TRUE),

    wall_ge4_n =
      sum(path_wall_ge4 == 1, na.rm = TRUE),

    wall_ge4_pct =
      100 * mean(path_wall_ge4 == 1, na.rm = TRUE)
  )

print(morph_summary, width = Inf)

c(
  transverse_UA_vs_EC =
    wilcox.test(
      UA$path_gb_transverse,
      EC$path_gb_transverse,
      exact = FALSE
    )$p.value,

  transverse_UA_vs_EA =
    wilcox.test(
      UA$path_gb_transverse,
      EA$path_gb_transverse,
      exact = FALSE
    )$p.value,

  wall_UA_vs_EC =
    wilcox.test(
      UA$path_wall_mm,
      EC$path_wall_mm,
      exact = FALSE
    )$p.value,

  wall_UA_vs_EA =
    wilcox.test(
      UA$path_wall_mm,
      EA$path_wall_mm,
      exact = FALSE
    )$p.value,

  wall_ge4_UA_vs_EC =
    fisher.test(
      table(
        c(rep("UA", nrow(UA)), rep("EC", nrow(EC))),
        c(UA$path_wall_ge4, EC$path_wall_ge4)
      )
    )$p.value,

  wall_ge4_UA_vs_EA =
    fisher.test(
      table(
        c(rep("UA", nrow(UA)), rep("EA", nrow(EA))),
        c(UA$path_wall_ge4, EA$path_wall_ge4)
      )
    )$p.value
)

# Preoperative radiological wall thickening vs pathological wall >=4 mm
elective_all <- analysis %>%
```

```
  filter(triage == 1)

table(
  Radiological = elective_all$wall_thickening,
  Pathological_ge4 = elective_all$path_wall_ge4
)

fit_rad_path <- glm(
  path_wall_ge4 ~ wall_thickening,
  data = elective_all,
  family = binomial
)

# Profile-likelihood 95% CI
exp(cbind(
  OR = coef(fit_rad_path),
  confint(fit_rad_path)
))

summary(fit_rad_path)$coefficients

# -------------------------------------------------------------
# 16. Three-group preoperative, operative, and postoperative
#     comparisons (Table 4)
# -------------------------------------------------------------
# Preoperative radiological wall thickening
fisher.test(
  table(
    factor(
      c(rep("UA", nrow(UA)), rep("EC", nrow(EC)))
    ),
    c(UA$wall_thickening, EC$wall_thickening)
  )
)

fisher.test(
  table(
    factor(
      c(rep("UA", nrow(UA)), rep("EA", nrow(EA)))
    ),
    c(UA$wall_thickening, EA$wall_thickening)
  )
)

# Distended/hydropic gallbladder
fisher.test(
  table(
    factor(
      c(rep("UA", nrow(UA)), rep("EC", nrow(EC)))
    ),
    c(UA$gb_distended, EC$gb_distended)
  )
)

fisher.test(
  table(
    factor(
      c(rep("UA", nrow(UA)), rep("EA", nrow(EA)))
    ),
    c(UA$gb_distended, EA$gb_distended)
  )
)

# Previous biliary attack
fisher.test(
  table(
    factor(
      c(rep("UA", nrow(UA)), rep("EC", nrow(EC)))
    ),
    c(UA$previous_attack, EC$previous_attack)
  )
)
```

```
fisher.test(
  table(
    factor(
      c(rep("UA", nrow(UA)), rep("EA", nrow(EA)))
    ),
    c(UA$previous_attack, EA$previous_attack)
  )
)

# CAR
wilcox.test(
  UA$car,
  EC$car,
  exact = FALSE
)

wilcox.test(
  UA$car,
  EA$car,
  exact = FALSE
)

# Elective waiting-list time
# Verify that elective waiting-list values converted cleanly
bad_waiting_values <- analysis %>%
  filter(
    triage == 1,
    !is.na(elective_wait_months),
    is.na(elective_wait_months_num)
  ) %>%
  select(id, elective_wait_months)

print(bad_waiting_values)

if (nrow(bad_waiting_values) > 0) {
  stop(
    "Non-numeric elective waiting-list values remain. ",
    "Please verify the source workbook before analysis."
  )
}

wilcox.test(
  UA$elective_wait_months_num,
  EC$elective_wait_months_num,
  exact = FALSE
)

# Operative time
wilcox.test(
  UA$operative_time,
  EC$operative_time,
  exact = FALSE
)

wilcox.test(
  UA$operative_time,
  EA$operative_time,
  exact = FALSE
)

# Documented omental adhesions
fisher.test(
  table(
    factor(
      c(rep("UA", nrow(UA)), rep("EC", nrow(EC)))
    ),
    c(UA$omental_adhesion, EC$omental_adhesion)
  )
)

fisher.test(
  table(
    factor(
```

```
      c(rep("UA", nrow(UA)), rep("EA", nrow(EA)))
    ),
    c(UA$omental_adhesion, EA$omental_adhesion)
  )
)

# Postoperative hospital stay >1 day
fisher.test(
  table(
    factor(
      c(rep("UA", nrow(UA)), rep("EC", nrow(EC)))
    ),
    c(UA$postop_stay_gt1, EC$postop_stay_gt1)
  )
)

fisher.test(
  table(
    factor(
      c(rep("UA", nrow(UA)), rep("EA", nrow(EA)))
    ),
    c(UA$postop_stay_gt1, EA$postop_stay_gt1)
  )
)

# 30-day complication
fisher.test(
  table(
    factor(
      c(rep("UA", nrow(UA)), rep("EC", nrow(EC)))
    ),
    c(UA$complication, EC$complication)
  )
)

fisher.test(
  table(
    factor(
      c(rep("UA", nrow(UA)), rep("EA", nrow(EA)))
    ),
    c(UA$complication, EA$complication)
  )
)

# Clavien-Dindo grade >=III
fisher.test(
  table(
    factor(
      c(rep("UA", nrow(UA)), rep("EC", nrow(EC)))
    ),
    c(UA$clavien_ge3, EC$clavien_ge3)
  )
)

fisher.test(
  table(
    factor(
      c(rep("UA", nrow(UA)), rep("EA", nrow(EA)))
    ),
    c(UA$clavien_ge3, EA$clavien_ge3)
  )
)

# -------------------------------------------------------------
# 17. Postoperative outcome models (Supplementary Table S6)
# -------------------------------------------------------------
elective_outcomes <- analysis %>%
  filter(
    group3 %in% c(
      "Unexpected acute",
      "Elective-chronic"
    )
  ) %>%
```

```
  mutate(
    UA = ifelse(
      group3 == "Unexpected acute",
      1,
      0
    )
  )

postop_summary <- elective_outcomes %>%
  group_by(group3) %>%
  summarise(
    n = n(),

    operative_time_median =
      median(operative_time, na.rm = TRUE),

    operative_time_q1 =
      quantile(operative_time, 0.25, na.rm = TRUE),

    operative_time_q3 =
      quantile(operative_time, 0.75, na.rm = TRUE),

    omental_n =
      sum(omental_adhesion == 1, na.rm = TRUE),

    omental_pct =
      100 * mean(omental_adhesion == 1, na.rm = TRUE),

    stay_gt1_n =
      sum(postop_stay_gt1 == 1, na.rm = TRUE),

    stay_gt1_pct =
      100 * mean(postop_stay_gt1 == 1, na.rm = TRUE),

    complication_n =
      sum(complication == 1, na.rm = TRUE),

    complication_pct =
      100 * mean(complication == 1, na.rm = TRUE)
  )

print(postop_summary, width = Inf)

fit_stay_unadj <- glm(
  postop_stay_gt1 ~ UA,
  data = elective_outcomes,
  family = binomial
)

fit_stay_ASA <- glm(
  postop_stay_gt1 ~ UA + asa,
  data = elective_outcomes,
  family = binomial
)

fit_stay_age_ASA <- glm(
  postop_stay_gt1 ~ UA + age + asa,
  data = elective_outcomes,
  family = binomial
)

fit_comp_unadj <- glm(
  complication ~ UA,
  data = elective_outcomes,
  family = binomial
)

fit_comp_ASA <- glm(
  complication ~ UA + asa,
  data = elective_outcomes,
  family = binomial
)
```

```
fit_comp_age_ASA <- glm(
  complication ~ UA + age + asa,
  data = elective_outcomes,
  family = binomial
)

extract_OR <- function(model, term) {
  beta <- coef(model)[term]
  se <- sqrt(vcov(model)[term, term])

  c(
    OR = exp(beta),
    lower_95_CI = exp(beta - 1.96 * se),
    upper_95_CI = exp(beta + 1.96 * se),
    p_value =
      summary(model)$coefficients[
        term,
        "Pr(>|z|)"
      ]
  )
}

rbind(
  Stay_unadjusted =
    extract_OR(fit_stay_unadj, "UA"),

  Stay_ASA =
    extract_OR(fit_stay_ASA, "UA"),

  Stay_age_ASA =
    extract_OR(fit_stay_age_ASA, "UA"),

  Complication_unadjusted =
    extract_OR(fit_comp_unadj, "UA"),

  Complication_ASA =
    extract_OR(fit_comp_ASA, "UA"),

  Complication_age_ASA =
    extract_OR(fit_comp_age_ASA, "UA")
)

# ------------------------------------------------------------
# 18. Two-feature stratification (Table 5)
# ------------------------------------------------------------
strat <- elective_cc %>%
  mutate(
    finding_count =
      previous_attack + wall_thickening
  )

table(strat$finding_count)

table5_summary <- strat %>%
  group_by(finding_count) %>%
  summarise(
    n = n(),

    unexpected_acute_n =
      sum(unexpected_acute == 1),

    unexpected_acute_pct =
      100 * mean(unexpected_acute == 1),

    operative_time_median =
      median(operative_time),

    operative_time_q1 =
      quantile(operative_time, 0.25),

    operative_time_q3 =
      quantile(operative_time, 0.75),
```

```
    omental_n =
      sum(omental_adhesion == 1),

    omental_pct =
      100 * mean(omental_adhesion == 1),

    stay_gt1_n =
      sum(postop_stay_gt1 == 1),

    stay_gt1_pct =
      100 * mean(postop_stay_gt1 == 1)
  )

print(table5_summary, width = Inf)

fit_factor_acute <- glm(
  unexpected_acute ~ finding_count,
  data = strat,
  family = binomial
)

fit_factor_oment <- glm(
  omental_adhesion ~ finding_count,
  data = strat,
  family = binomial
)

fit_factor_yatis <- glm(
  postop_stay_gt1 ~ finding_count,
  data = strat,
  family = binomial
)

extract_OR(
  fit_factor_acute,
  "finding_count"
)

extract_OR(
  fit_factor_oment,
  "finding_count"
)

extract_OR(
  fit_factor_yatis,
  "finding_count"
)

cor.test(
  strat$finding_count,
  strat$operative_time,
  method = "spearman",
  exact = FALSE
)

# Compare linear and categorical specifications
fit_acute_linear <- glm(
  unexpected_acute ~ finding_count,
  data = strat,
  family = binomial
)

fit_acute_cat <- glm(
  unexpected_acute ~ factor(finding_count),
  data = strat,
  family = binomial
)

fit_oment_linear <- glm(
  omental_adhesion ~ finding_count,
  data = strat,
  family = binomial
)
```

```
fit_oment_cat <- glm(
  omental_adhesion ~ factor(finding_count),
  data = strat,
  family = binomial
)

fit_stay_linear <- glm(
  postop_stay_gt1 ~ finding_count,
  data = strat,
  family = binomial
)

fit_stay_cat <- glm(
  postop_stay_gt1 ~ factor(finding_count),
  data = strat,
  family = binomial
)

anova(
  fit_acute_linear,
  fit_acute_cat,
  test = "LRT"
)

anova(
  fit_oment_linear,
  fit_oment_cat,
  test = "LRT"
)

anova(
  fit_stay_linear,
  fit_stay_cat,
  test = "LRT"
)
# Final p values for linear vs categorical specification:
# unexpected acute = 0.729
# documented omental adhesions = 0.210
# postoperative hospital stay >1 day = 0.788

# ------------------------------------------------------------
# 19. Chronic-only stratification (Table 6)
# ------------------------------------------------------------
chronic_strat <- strat %>%
  filter(acute_pathology == 0)

nrow(chronic_strat)
table(chronic_strat$finding_count)

table6_summary <- chronic_strat %>%
  group_by(finding_count) %>%
  summarise(
    n = n(),

    operative_time_median =
      median(operative_time),

    operative_time_q1 =
      quantile(operative_time, 0.25),

    operative_time_q3 =
      quantile(operative_time, 0.75),

    omental_n =
      sum(omental_adhesion == 1),

    omental_pct =
      100 * mean(omental_adhesion == 1),

    stay_gt1_n =
      sum(postop_stay_gt1 == 1),
```

```
    stay_gt1_pct =
      100 * mean(postop_stay_gt1 == 1)
  )

print(table6_summary, width = Inf)

fit_CH_oment <- glm(
  omental_adhesion ~ finding_count,
  data = chronic_strat,
  family = binomial
)

fit_CH_yatis <- glm(
  postop_stay_gt1 ~ finding_count,
  data = chronic_strat,
  family = binomial
)

extract_OR(
  fit_CH_oment,
  "finding_count"
)

extract_OR(
  fit_CH_yatis,
  "finding_count"
)

cor.test(
  chronic_strat$finding_count,
  chronic_strat$operative_time,
  method = "spearman",
  exact = FALSE
)

fisher.test(
  table(
    chronic_strat$finding_count,
    chronic_strat$omental_adhesion
  )
)

# -------------------------------------------------------------
# 20. Sensitivity analysis S9:
#     Excluding acute-on-chronic cholecystitis
# -------------------------------------------------------------
sens_excl <- analysis %>%
  filter(
    histology_code != 3,
    triage == 1,
    !is.na(previous_attack),
    !is.na(wall_thickening),
    !is.na(car)
  ) %>%
  mutate(
    unexpected_acute_s9 = ifelse(
      histology_code %in% c(1, 4, 5),
      1,
      0
    )
  )

fit_SA <- glm(
  unexpected_acute_s9 ~
    previous_attack +
    wall_thickening,
  data = sens_excl,
  family = binomial
)

fit_SB <- glm(
  unexpected_acute_s9 ~
    previous_attack +
```

```
    wall_thickening +
    car,
  data = sens_excl,
  family = binomial
)

exp(cbind(
  OR = coef(fit_SA),
  confint.default(fit_SA)
))

exp(cbind(
  OR = coef(fit_SB),
  confint.default(fit_SB)
))

beta_s9 <- coef(fit_SB)["car"]
se_s9 <- sqrt(
  vcov(fit_SB)["car", "car"]
)

c(
  CAR_OR_per_5_units =
    exp(5 * beta_s9),

  lower_95_CI =
    exp(
      5 *
        (beta_s9 - 1.96 * se_s9)
    ),

  upper_95_CI =
    exp(
      5 *
        (beta_s9 + 1.96 * se_s9)
    ),

  p_value =
    summary(fit_SB)$coefficients[
      "car",
      "Pr(>|z|)"
    ]
)

pred_SA <- predict(
  fit_SA,
  type = "response"
)

pred_SB <- predict(
  fit_SB,
  type = "response"
)

roc_SA <- pROC::roc(
  sens_excl$unexpected_acute_s9,
  pred_SA,
  quiet = TRUE
)

roc_SB <- pROC::roc(
  sens_excl$unexpected_acute_s9,
  pred_SB,
  quiet = TRUE
)

c(
  AUC_A = as.numeric(pROC::auc(roc_SA)),
  AUC_B = as.numeric(pROC::auc(roc_SB)),
  AIC_A = AIC(fit_SA),
  AIC_B = AIC(fit_SB)
)
```

```
anova(
  fit_SA,
  fit_SB,
  test = "LRT"
)

# ------------------------------------------------------------
# 21. Sensitivity analysis S10:
#     Acute-on-chronic reclassified as chronic
# ------------------------------------------------------------
sens_reclass <- analysis %>%
  filter(
    triage == 1,
    !is.na(previous_attack),
    !is.na(wall_thickening),
    !is.na(car)
  ) %>%
  mutate(
    unexpected_acute_s10 = ifelse(
      histology_code %in% c(1, 4, 5),
      1,
      0
    )
  )

fit_R2A <- glm(
  unexpected_acute_s10 ~
    previous_attack +
    wall_thickening,
  data = sens_reclass,
  family = binomial
)

fit_R2B <- glm(
  unexpected_acute_s10 ~
    previous_attack +
    wall_thickening +
    car,
  data = sens_reclass,
  family = binomial
)

c(
  AIC_A = AIC(fit_R2A),
  AIC_B = AIC(fit_R2B)
)

anova(
  fit_R2A,
  fit_R2B,
  test = "LRT"
)

# ------------------------------------------------------------
# 22. Exploratory analysis of gangrenous cholecystitis
# Final reproducible values: AUC 0.684; bootstrap 95% CI 0.588-0.777;
# Youden threshold 5.03; sensitivity 88.5%; specificity 49.6%
#     (Supplementary Table S11)
# ------------------------------------------------------------
acute_cases <- analysis %>%
  filter(acute_pathology == 1) %>%
  mutate(
    gangrenous =
      ifelse(histology_code == 5, 1, 0)
  )

table(acute_cases$gangrenous)

gangrenous_summary <- acute_cases %>%
  group_by(gangrenous) %>%
  summarise(
    n = n(),
```

```
    CAR_median =
      median(car, na.rm = TRUE),

    CAR_q1 =
      quantile(car, 0.25, na.rm = TRUE),

    CAR_q3 =
      quantile(car, 0.75, na.rm = TRUE)
  )

print(
  gangrenous_summary,
  width = Inf
)

wilcox.test(
  car ~ gangrenous,
  data = acute_cases,
  exact = FALSE
)

roc_gang <- pROC::roc(
  response = acute_cases$gangrenous,
  predictor = acute_cases$car,
  quiet = TRUE
)

as.numeric(
  pROC::auc(roc_gang)
)

set.seed(2026)

ci_gang <- pROC::ci.auc(
  roc_gang,
  method = "bootstrap",
  boot.n = 2000,
  boot.stratified = TRUE
)

ci_gang

youden <- pROC::coords(
  roc_gang,
  x = "best",
  best.method = "youden",
  ret = c(
    "threshold",
    "sensitivity",
    "specificity"
  ),
  transpose = FALSE
)

youden

# Emphysematous cases included in the non-gangrenous comparator
sum(
  acute_cases$histology_code == 4
)

# -----------------------------------------------------------
# 23. Missing-data and complication checks
# -----------------------------------------------------------
# Missing-data summary for key analytical variables
missing_summary <- data.frame(
  Variable = c(
    "Histopathological diagnosis",
    "Surgical triage",
    "Previous biliary attack history",
    "C-reactive protein/albumin ratio",
    "Gallbladder wall thickening",
    "Gallbladder distension",
```

```
    "Operative time",
    "Documented omental adhesions",
    "Postoperative hospital stay",
    "30-day complication",
    "Clavien-Dindo grade"
  ),

  Missing_n = c(
    sum(is.na(analysis$histology_code)),
    sum(is.na(analysis$triage)),
    sum(is.na(analysis$previous_attack)),
    sum(is.na(analysis$car)),
    sum(is.na(analysis$wall_thickening)),
    sum(is.na(analysis$gb_distended)),
    sum(is.na(analysis$operative_time)),
    sum(is.na(analysis$omental_adhesion)),
    sum(is.na(analysis$hospital_stay)),
    sum(is.na(analysis$complication)),
    sum(is.na(analysis$clavien))
  )
)

missing_summary$Missing_percent <-
  100 *
  missing_summary$Missing_n /
  nrow(analysis)

missing_summary

# Complication types among patients with a 30-day complication
table(
  analysis$complication_type[
    analysis$complication == 1
  ],
  useNA = "ifany"
)

# Clavien-Dindo distribution among patients with a complication
table(
  analysis$clavien[
    analysis$complication == 1
  ],
  useNA = "ifany"
)

# Total complications and grade >=III
c(
  total_30_day_complications =
    sum(analysis$complication == 1, na.rm = TRUE),

  grade_ge3 =
    sum(analysis$clavien_ge3 == 1, na.rm = TRUE)
)

# Major bile duct injuries
bdi_cases <- analysis %>%
  filter(
    complication_type == 6
  ) %>%
  select(
    id,
    triage,
    acute_pathology,
    complication_treatment,
    clavien,
    group3
  )

bdi_cases
table(bdi_cases$group3)

# ------------------------------------------------------------
# 24. Session information
```

```
# ------------------------------------------------------------
sessionInfo()
```
