# Supplementary material for: Clinical–Histopathological Concordance of Surgical Triage and the Unexpected Acute Phenotype in 773 Consecutive Laparoscopic Cholecystectomies
Source: Medicina (Kaunas). 2026 Oct 16;62(10):2003. doi: 10.3390/medicina62102003 (PMC13707155; doi:10.3390/medicina62102003)
Supplement: Supplementary file 1 [file medicina-62-02003-s001.zip › supplementary information.pdf]

**Supplementary Table S1. Variable coding dictionary**

| Variable | Coding |
|---|---|
| Sex | 0=female; 1=male |
| Surgical triage | 0=emergency; 1=elective |
| Radiological gallbladder wall | 1=normal; 2=thickened |
| Radiological gallbladder size | 1=normal; 2=distended/hydropic |
| Previous biliary attack | 0=no; 1=yes; 9=unknown |
| Main histopathological diagnosis | 1=acute cholecystitis; 2=chronic cholecystitis; 3=acute-on-chronic cholecystitis; 4=emphysematous cholecystitis; 5=gangrenous cholecystitis; 6=adenocarcinoma; 7=xanthogranulomatous chronic cholecystitis; 8=eosinophilic calculous cholecystitis |
| Postoperative complication | 0=no; 1=yes |
| Complication type | 1=bile fistula; 2=surgical-site infection; 3=postoperative pancreatitis; 4=postoperative cholangitis/common bile duct stone; 5=gallbladder-bed abscess/collection; 6=bile duct injury; 7=postoperative hemorrhage |
| Complication treatment | 1=medical treatment/wound care; 2=ERCP; 3=surgical treatment; 4=percutaneous/interventional treatment |
| Clavien–Dindo grade | 0=no complication; 1=grade I; 2=grade II; 3=grade IIIa; 5=grade IIIb |
| Acute symptom-to-surgery interval | 0=0–72 h; 1=72 h–1 week; 2=7–10 days |

Unknown previous-attack history (code 9) was treated as missing in analyses involving that variable. ERCP, endoscopic retrograde cholangiopancreatography. Coding definitions were based on the original study data dictionary.

**Supplementary Table S2. Study population and histopathological diagnosis distribution**

**S2A. Patient flow**

| Stage | n |
|---|---|
| Consecutive patients whose cholecystectomy was completed laparoscopically | 780 |
| Excluded: adenocarcinoma | 4 |
| Excluded: eosinophilic calculous cholecystitis | 3 |
| Final analytical population | 773 |

Adenocarcinoma and eosinophilic calculous cholecystitis were excluded because they could not be appropriately classified within the predefined acute or chronic inflammatory pathology categories.

**S2B. Histopathological diagnosis distribution**

| Histopathological diagnosis | n | Classification in the primary analysis |
|---|---|---|
| Chronic cholecystitis | 602 | Chronic inflammatory pathology |
| Acute cholecystitis | 107 | Acute inflammatory pathology |
| Acute-on-chronic cholecystitis | 27 | Acute inflammatory pathology |
| Gangrenous cholecystitis | 26 | Acute inflammatory pathology |
| Xanthogranulomatous chronic cholecystitis | 8 | Chronic inflammatory pathology |
| Emphysematous cholecystitis | 3 | Acute inflammatory pathology |
| Acute inflammatory pathology, total | 163 | — |
| Chronic inflammatory pathology, total | 610 | — |
| Total | 773 | — |

Acute-on-chronic cholecystitis was classified within the acute inflammatory pathology category in the primary analysis. The robustness of this classification was examined in two sensitivity analyses: exclusion of acute-on-chronic cases and reclassification of these cases into the chronic inflammatory pathology category.

**Supplementary Table S3. Missing-data summary**

| Variable | Missing, n | Missing, % | Handling |
|---|---|---|---|
| Histopathological diagnosis | 0 | 0.0 | — |
| Surgical triage | 0 | 0.0 | — |
| Previous biliary attack history | 11 | 1.4 | Unknown values treated as missing; complete-case analysis |
| C-reactive protein/albumin ratio | 0 | 0.0 | — |
| Gallbladder wall thickening | 0 | 0.0 | — |
| Gallbladder distension | 0 | 0.0 | — |
| Operative time | 0 | 0.0 | — |
| Documented omental adhesions | 0 | 0.0 | — |
| Postoperative hospital stay | 0 | 0.0 | — |
| 30-day complication | 0 | 0.0 | — |
| Clavien–Dindo grade | 0 | 0.0 | — |

Time on the elective waiting list was defined only for electively scheduled patients; values were therefore structurally undefined, rather than missing, in emergency patients.

**Supplementary Table S4. Detailed comparison of Model A and Model B in the primary elective cohort**

| Measure | Model A | Model B |
|---|---|---|
| Patients / events | 627 / 42 | 627 / 42 |
| Events per variable | 21.0 | 14.0 |
| AUC | 0.697 | 0.756 |
| Bootstrap 95% CI for AUC | 0.607–0.780 | 0.673–0.838 |
| Bootstrap optimism for AUC | 0.001 | 0.004 |
| Optimism-corrected AUC | 0.696 | 0.752 |
| AIC | 282.50 | 272.57 |
| Brier score | 0.0576 | 0.0548 |
| Likelihood-ratio test | — | $p$=0.00055 |
| Optimism-corrected calibration intercept | 0.012 | 0.009 |
| Optimism-corrected calibration slope | 1.013 | 0.942 |

Model A included previous biliary attack and preoperative radiological gallbladder wall thickening. Model B additionally included CAR. AUC confidence intervals were estimated using 2,000 bootstrap resamples, with the model refitted within each bootstrap sample. Internal validation was performed using 500 bootstrap resamples for optimism correction. Calibration intercept and slope were internally optimism-corrected using 500 bootstrap resamples; ideal values are 0 and 1, respectively. Addition of CAR improved model fit and discrimination, although its absolute contribution to individual predicted probabilities was modest relative to the two binary clinical findings.

**Supplementary Table S5. Predicted probability of unexpected acute pathology according to CAR level and two binary clinical findings**

| Previous biliary attack | Preoperative radiological gallbladder wall thickening | CAR at median (0.65) | CAR at 75th percentile (1.29) | CAR at 90th percentile (2.74) |
|---|---|---|---|---|
| No | No | 3.5% | 3.7% | 4.1% |
| Yes | No | 9.0% | 9.5% | 10.6% |
| No | Yes | 12.8% | 13.4% | 14.9% |
| Yes | Yes | 28.9% | 30.0% | 32.6% |

Predicted probabilities were derived from Model B. Although CAR improved overall model discrimination and fit, increasing CAR from the median to the 90th percentile produced only a modest absolute increase in predicted probability within each clinical stratum. CAR should therefore be interpreted as a complementary biomarker rather than the primary stratification variable.

**Supplementary Table S6. Sensitivity analyses of postoperative outcomes adjusted for ASA score and age**

**S6A. Postoperative hospital stay >1 day**

| **Model** | **OR** | **95% CI** | ***p* value** |
|---|---|---|---|
| Unadjusted | 5.18 | 2.64–10.17 | <0.001 |
| Adjusted for ASA score | 6.61 | 3.10–14.10 | <0.001 |
| Adjusted for age and ASA score | 6.63 | 3.04–14.45 | <0.001 |

**S6B. 30-day postoperative complication**

| **Model** | **OR** | **95% CI** | ***p* value** |
|---|---|---|---|
| Unadjusted | 2.87 | 0.94–8.77 | 0.065 |
| Adjusted for ASA score | 2.93 | 0.88–9.71 | 0.080 |
| Adjusted for age and ASA score | 2.77 | 0.83–9.26 | 0.097 |

The reference group comprised electively scheduled patients with chronic histopathology. The association between unexpected acute pathology and postoperative hospital stay >1 day remained robust after adjustment for age and ASA score. The association with 30-day postoperative complications did not reach statistical significance in any model.

**Supplementary Table S7. Detailed distribution of postoperative complications**

**S7A. Complication type**

| **Complication** | **Code** | **n** |
|---|---|---|
| Bile fistula | 1 | 19 |
| Surgical-site infection | 2 | 6 |
| Postoperative pancreatitis | 3 | 7 |
| Postoperative cholangitis/common bile duct stone | 4 | 9 |
| Gallbladder-bed abscess/collection | 5 | 2 |
| Bile duct injury | 6 | 3 |
| Postoperative hemorrhage | 7 | 1 |
| Total | — | 47 |

**S7B. Clavien–Dindo distribution**

| **Clavien–Dindo grade** | **n** |
|---|---|
| Grade I | 10 |
| Grade II | 21 |
| Grade IIIa | 13 |
| Grade IIIb | 3 |
| Grade IV | 0 |
| Grade V | 0 |
| Grade ≥III | 16 |
| 30-day mortality | 0 |

Complication categories and Clavien–Dindo grades were verified against the original study data dictionary. Bile fistula and major bile duct injury were coded as separate complication categories.

**Supplementary Table S8. Major bile duct injuries requiring intervention**

| Case | Surgical triage | Histopathological classification | Treatment | Clavien–Dindo grade |
|---|---|---|---|---|
| 1 | Emergency | Acute inflammatory pathology | Surgical repair | IIIb |
| 2 | Emergency | Acute inflammatory pathology | ERCP with stenting | IIIa |
| 3 | Elective | Chronic inflammatory pathology | Surgical repair | IIIb |

Major bile duct injury occurred in 3/773 patients (0.4%). Two cases occurred in the emergency-acute group and one in the elective-chronic group. No major bile duct injury occurred in the unexpected acute subgroup. ERCP, endoscopic retrograde cholangiopancreatography.

**Supplementary Table S9. Sensitivity analysis excluding acute-on-chronic cholecystitis**

In this sensitivity analysis, patients with acute-on-chronic cholecystitis were excluded from the analysis.

**S9A. Multivariable associations**

| Variable | Model A OR (95% CI) | Model B OR (95% CI) | Model B *p* value |
|---|---|---|---|
| Previous biliary attack | 3.48 (1.61–7.54) | 3.06 (1.35–6.93) | 0.007 |
| Preoperative radiological gallbladder wall thickening | 5.25 (2.28–12.13) | 4.29 (1.75–10.57) | 0.002 |
| C-reactive protein/albumin ratio, per 5-unit increase | — | 1.61 (1.26–2.06) | <0.001 |

**S9B. Model performance**

| Measure | Model A | Model B |
|---|---|---|
| AUC | 0.719 | 0.798 |
| AIC | 223.52 | 211.38 |
| Likelihood-ratio test for addition of CAR | — | $p$=0.00017 |

Model A included previous biliary attack and preoperative radiological gallbladder wall thickening. Model B additionally included the C-reactive protein/albumin ratio (CAR). Exclusion of acute-on-chronic cholecystitis did not attenuate the associations observed in the primary analysis.

**Supplementary Table S10. Sensitivity analysis reclassifying acute-on-chronic cholecystitis into the chronic inflammatory pathology category**

| Measure | Model A | Model B |
|---|---|---|
| AIC | 225.96 | 213.89 |
| Likelihood-ratio test for addition of CAR | — | $p$=0.00018 |

In this sensitivity analysis, acute-on-chronic cholecystitis was retained in the analytical cohort but reclassified within the chronic inflammatory pathology category. The addition of CAR continued to improve model fit, supporting its incremental contribution under this alternative histopathological classification.

**Supplementary Table S11. Exploratory performance of CAR for identifying gangrenous cholecystitis**

| **Measure** | **Value** |
|---|---|
| Patients with acute inflammatory pathology | 163 |
| Gangrenous cholecystitis | 26/163 (16.0%) |
| CAR, gangrenous cases | 21.2 (9.7–52.2) |
| CAR, other acute inflammatory pathologies | 5.1 (1.4–32.9) |
| Mann–Whitney U test | $p$=0.003 |
| AUC | 0.684 |
| Bootstrap 95% CI | 0.588–0.777 |
| Data-derived Youden threshold | 5.03 |
| Sensitivity | 88.5% |
| Specificity | 49.6% |

This analysis was exploratory. The threshold was derived from the same cohort and was not externally validated; therefore, it should not be interpreted as a clinical decision threshold. The limited specificity further precludes use of CAR as a standalone marker for gangrenous cholecystitis. The three patients with emphysematous cholecystitis were included among the other non-gangrenous acute inflammatory pathologies; no separate analysis was performed because of the small number of cases.
